# Antimicrobial and Immunomodulating Activities of Two Endemic *Nepeta* Species and Their Major Iridoids Isolated from Natural Sources

**DOI:** 10.3390/ph14050414

**Published:** 2021-04-28

**Authors:** Neda Aničić, Uroš Gašić, Feng Lu, Ana Ćirić, Marija Ivanov, Bojan Jevtić, Milena Dimitrijević, Boban Anđelković, Marijana Skorić, Jasmina Nestorović Živković, Yingle Mao, Jia Liu, Chunping Tang, Marina Soković, Yang Ye, Danijela Mišić

**Affiliations:** 1 Department of Plant Physiology, Institute for Biological Research “Siniša Stanković”–National Institute of Republic of Serbia, University of Belgrade, Bulevar Despota Stefana 142, 11060 Belgrade, Serbia; neda.anicic@ibiss.bg.ac.rs (N.A.); rancic@ibiss.bg.ac.rs (A.Ć.); marija.smiljkovic@ibiss.bg.ac.rs (M.I.); mdevic@ibiss.bg.ac.rs (M.S.); jasmina.nestorovic@ibiss.bg.ac.rs (J.N.Ž.); mris@ibiss.bg.ac.rs (M.S.); 2 State Key Laboratory of Drug Research and Department of Natural Product Chemistry, Shanghai Institute of Materia Medica, Chinese Academy of Sciences, 555 Zu-Chong-Zhi Road, Zhangjiang Hi-Tech Park, Shanghai 201203, China; fenglu@simm.ac.cn (F.L.); maoyingle@simm.ac.cn (Y.M.); jia.liu@simm.ac.cn (J.L.); tangcp@simm.ac.cn (C.T.); yye@simm.ac.cn (Y.Y.); 3 Department of Immunology, Institute for Biological Research “Siniša Stanković”–National Institute of Republic of Serbia, University of Belgrade, Bulevar Despota Stefana 142, 11060 Belgrade, Serbia; bojanbh@gmail.com; 4 Center of Excellence for Green Technologies, Institute for Multidisciplinary Research, University of Belgrade, Kneza Višeslava 1, 11030 Belgrade, Serbia; milena.dimitrijevic@imsi.rs; 5 Faculty of Chemistry, University of Belgrade, P.O. Box 51, 11000 Belgrade, Serbia; aboban@chem.bg.ac.rs

**Keywords:** *Nepeta*, nepetalactone, 1,5,9-epideoxyloganic acid, antimicrobial activity, antibiofilm, immunomodulatory effects

## Abstract

Two Balkan Peninsula endemics, *Nepeta rtanjensis* and *N. argolica* subsp. *argolica*, both characterized by specialized metabolite profiles predominated by iridoids and phenolics, are differentiated according to the stereochemistry of major iridoid aglycone nepetalactone (NL). For the first time, the present study provides a comparative analysis of antimicrobial and immunomodulating activities of the two *Nepeta* species and their major iridoids isolated from natural sources—*cis,trans*-NL, *trans,cis*-NL, and 1,5,9-epideoxyloganic acid (1,5,9-*e*DLA), as well as of phenolic acid rosmarinic acid (RA). Methanol extracts and pure iridoids displayed excellent antimicrobial activity against eight strains of bacteria and seven strains of fungi. They were especially potent against food-borne pathogens such as *L. monocytogenes*, *E. coli*, *S. aureus*, *Penicillium* sp., and *Aspergillus* sp. Targeted iridoids were efficient agents in preventing biofilm formation of resistant *P. aeruginosa* strain, and they displayed additive antimicrobial interaction. Iridoids are, to a great extent, responsible for the prominent antimicrobial activities of the two *Nepeta* species, although are probably minor contributors to the moderate immunomodulatory effects. The analyzed iridoids and RA, individually or in mixtures, have the potential to be used in the pharmaceutical industry as potent antimicrobials, and in the food industry to increase the shelf life and safety of food products.

## 1. Introduction

The plant genus *Nepeta* L. (family Lamiaceae) comprises around 280 species native to Europe, Asia, and Africa, commonly known as catmints or catnips. These remarkable plants are widely used in folk medicine against a variety of diseases and disorders, as well as in traditional food production [1], and their antimicrobial potential against human pathogenic microorganisms and phytopathogens has been comprehensively documented [2,3,4]. The majority of reported studies highlight monoterpenoid iridoid nepetalactone (NL) as the main bioactive compound in *Nepeta* sp.

Nepetalactone is usually present in the form of 7S diastereoisomers, which can be found in species-specific amounts and ratios [1,5]. Some studies proved that, even though NL isomers differ only in the orientation of a single chemical bond, they still show differential bioactivities, including attractant effects on cats [6], sex pheromone activity in some aphids [7], as well as repellent activities against a variety of insects [8,9,10]. Antimicrobial activities of pure nepetalactones have only rarely been tested [3,11], and differential activity of the two NL isomers was confirmed against *Helicobacter pylori* [3]. On the other hand, *Nepeta* species are also a rich source of iridoid glucosides [12,13,14,15], which have been unjustifiably neglected and only rarely studied for their biological activities. A literature survey for data on the bioactivities of one of the most abundant iridoid compounds in *Nepeta* species, 1,5,9-epideoxyloganic acid (1,5,9-*e*DLA), revealed no results.

Thus, the present study aimed to isolate *trans*,*cis*-NL, *cis*,*trans*-NL, and 1,5,9-*e*DLA from natural sources, and to comparatively analyze, for the first time, their antimicrobial activities with those of the crude methanol extracts of the two rare *Nepeta* species: (1) *Nepeta rtanjensis* Diklić & Milojević, a critically endangered perennial of Serbia, which is characterized by high amounts of *trans*,*cis*-NL, dehydronepetalactone (DNL), and 1,5,9-*e*DLA, while *cis*,*trans*-NL is present as a minor constituent [4,16,17,18]; (2) Greek endemic *N. argolica* Bory & Chaub. subsp. *argolica* (syn. *N. sibthorpii* Bentham, Euro + Med PlantBase), a *cis*,*trans*-NL-rich chemotype [17,18]. Antimicrobial activity was tested in microdilution assays against a series of food-borne and disease-causing bacteria and fungi. To supplement the knowledge on the mode of antimicrobial action, anti-biofilm activity and immunomodulatory effects of methanol extracts of the two model species and isolated compounds were analyzed. For the first time, antimicrobial interaction of targeted iridoid compounds and rosmarinic acid (RA) has been investigated within the present study.

## 2. Results and Discussion

Food-borne illnesses continue to be a frequent and severe threat to public health worldwide. Most food-borne diseases originate from bacterial or fungal pathogens that have contaminated the food at some point along the food chain from farm to fork. As the epidemiology of food-borne illnesses changes, with newly recognized pathogens emerging and well-recognized pathogens becoming associated with new food products, a growing number of synthetic additives and antibiotics are used to control infections. Given that the safety of synthetic antimicrobials has been questioned in recent years and that consumers demand more “green/organic” products, the imminent future of alternative food preservatives could be in bioactive molecules of plant origin.

*Nepeta* species are a promising source of effective antimicrobial agents for the food and pharmaceutical industries. The high content of monoterpenoid iridoids and phenolic compounds in *Nepeta* species is chiefly responsible for their prominent antimicrobial potential [3,4,16,19]. Although iridoid aglycones nepetalactones are highlighted as foremost responsible for the recorded bioactivities, iridoid glucosides, which represent significant contributors to the iridoids pool of *Nepeta* species, should not be neglected.

The present study aimed to test the bioactivity of two *Nepeta* species differing in the stereochemistry of major nepetalactones, *N. rtanjensis* and *N. argolica* subsp. *argolica*, and of their major bioactive principles. For this purpose, *cis,trans*-NL, *trans,cis*-NL, and 1,5,9-*e*DLA were isolated from natural sources, and their structures were determined by extensive analysis of NMR and mass data, as well as by comparison with those in the literature.

### 2.1. Isolation of cis,trans- and trans,cis-Nepetalactone

**Figure 1:**
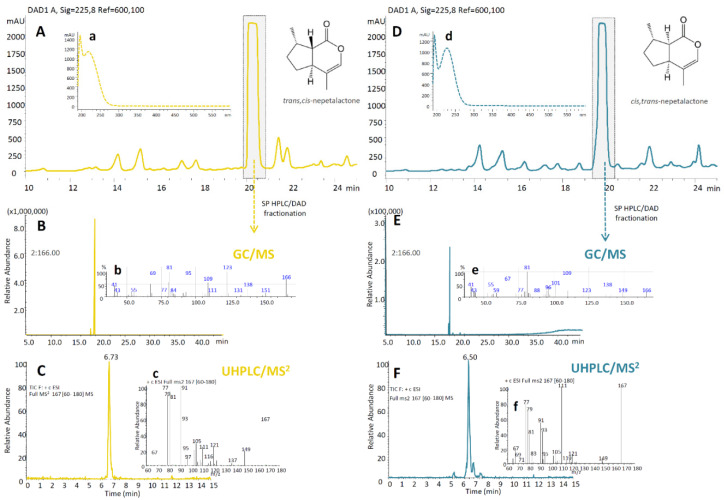
Semipreparative HPLC/DAD chromatograms of *Nepeta rtanjensis* (**A**) and *N. cataria* (**D**) essential oils (EO) acquired at λ = 230 nm, and corresponding UV spectra of *trans,cis*-nepetalactone (a) and *cis,trans*–nepetalactone (b). Isolated nepetalactones were subjected to structural characterization adopting GC/MS (**B**,**E**) and UHPLC/MS^2^ (**C**,**F**) analyses. Corresponding GC/MS (b,e) and MS^2^ spectra (c,f) are also presented.

Semi-preparative HPLC/DAD chromatograms of *N. rtanjensis* EO revealed *trans,cis*-NL eluting at Rt = 20.263 min (Figure 1A) and displaying absorption spectra with λ_max_ = 225 nm (Figure 1a). *cis,trans*-NL was visible as a peak at the *N. cataria* EO HPLC/DAD chromatogram eluting at Rt = 19.764 min (Figure 1D), and also showing λ_max_ = 225 nm (Figure 1d). Fractions containing *cis,trans*-NL (in 69% MeOH), and *trans,cis*-NL (70% MeOH) were separately combined, extracted with n-hexane, and evaporated to dryness at room temperature. The total yield of *cis,trans*-NL was 2 mg, and of *trans,cis*-NL it was 12 mg. A similar strategy to isolate NL stereoisomers from *N. cataria* EOs was previously adopted by Wang et al. [20].

To confirm the identification of *cis,trans*-NL and *trans,cis*-NL, collected fractions were subjected to GC/MS analysis. The *cis,trans-*NL eluted at Rt = 18.837 min (Figure 1B), while *trans,cis*-NL was visible as a peak at Rt = 18.043 min on the GC/MS chromatogram (Figure 1E). GC/MS spectra of the two NL stereoisomers showed the similar fragmentation patterns, with slightly different intensities of the major daughter ions (Figure 1b,e).

Further characterization of *cis,trans*- and *trans,cis*-NL was performed, adopting a UHPLC/DAD/(+)HESI−MS^2^ instrument. The *trans,cis*-NL was visible on the UHPLC/MS^2^ chromatogram as the molecular ion [M+H]^+^ at *m/z* 167, which eluted at Rt = 6.73 min (Figure 1C). *cis,trans*-NL was identified as the molecular ion [M+H]^+^ at *m/z* 167, eluting at Rt = 6.50 min (Figure 1F). The product ion scanning (PIS) experiment of the MS instrument revealed that the two NL diastereoisomers showed similar (+)MS^2^ fragmentation patterns, although the intensity of the fragments varied (Figure 1c,d), similarly as in Mišić et al. [17].

An unambiguous assignment of the NMR spectra of the two NL stereoisomers has been achieved using 1D (^1^H and ^13^C) and 2D (COSY, NOESY and HSQC) NMR techniques, and the results are shown in Supplementary Figures S1 and S2.

### 2.2. Isolation of 1,5,9-Epideoxyloganic Acid

**Figure 2:**
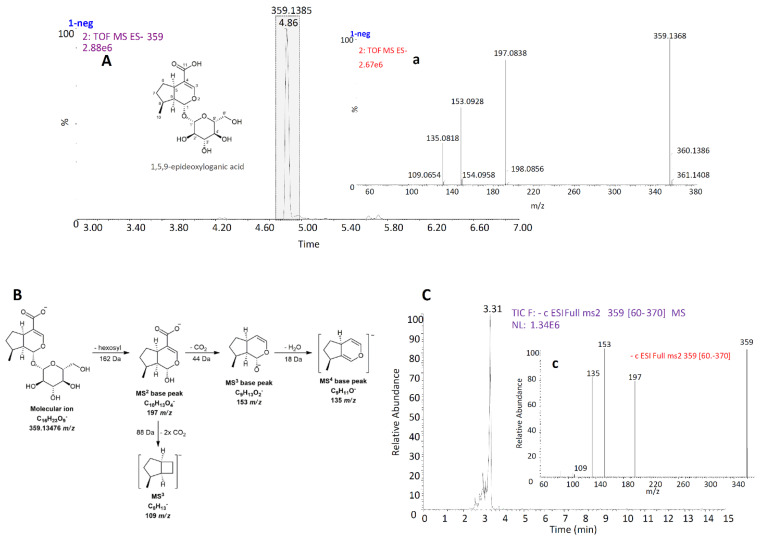
UPLC/Q-TOF MS chromatogram of *N. rtanjensis* methanol extract (**A**), with extracted mass for pseudomolecular ion [M−H]^−^ at *m/z* 359, corresponding MS^2^ spectrum of 1,5,9-epideoxyloganic acid (a), and proposed fragmentation pathway (**B**). Following preparative isolation of 1,5,9-epideoxyloganic acid from methanol extract, its structural characterization included UHPLC/MS^2^ analysis. UHPLC/qqqMS^2^ chromatogram (**C**) and corresponding (−)MS^2^ spectrum (c) are presented.

Iridoid glucoside, 1,5,9-*e*DLA, was isolated from a methanol extract of *N. rtanjensis*. UPLC/Q-TOF MS analysis of the extract revealed that 1,5,9-*e*DLA, eluting at Rt = 4.86 min (Figure 2A), was visible in a negative mode of the instrument, and it displayed the pseudomolecular ion [M−H]^−^ at *m/z* 359.1368. Its MS^2^ fragmentation pattern (Figure 2a) revealed a base peak at 197 *m/z* (loss of hexose, 162 Da) and MS^3^ base peak at 153 *m/z* (further loss of CO_2_, 44 Da). The loss of another molecule of CO_2_ resulted in a secondary MS^3^ peak at 109 *m/z*. Finally, fragmentation of the MS^3^ base peak (153 *m/z*) resulted in an MS^4^ base peak (135 *m/z*) by the loss of one water molecule (18 Da). The proposed fragmentation pattern of 1,5,9-*e*DLA is presented in Figure 2B.

Structural characterization of the isolated compound was performed using UHPLC/DAD/(+)HESI−MS^2^ (Figure 2C(c)) and NMR analysis (Supplementary Figure S3). NMR data almost resembled those reported in the literature [12,15]. The total yield of 1,5,9-*e*DLA was 133 mg.

### 2.3. Chemical Composition of N. rtanjensis and N. argolica Subsp. argolica Methanol Extracts

According to the literature, the extracts of plants of the genus *Nepeta* contain a wide variety of phytochemicals; the most common are monoterpenoids, among which glycosides and aglycones of iridoids predominate [5,21]. Phenolics from the groups of phenolic acids and flavonoids, both aglycones and glycosides, are also very abundant in *Nepeta* species [5,17,19].

The present study gives insight into the profile of polyphenolics and iridoids of *Nepeta argolica* subsp. *argolica* and *N. rtanjensis* methanol extracts using non-targeted and targeted metabolomics approaches. UHPLC-LTQ OrbiTrap XL fingerprinting resulted in identifying 49 compounds (Table 1) in analyzed samples. The tentative identification of compounds was based on high-resolution mass spectrometry (HRMS) and MS^4^ fragmentation, as well as literature data [13,17,22,23]. All identified compounds could be divided into four major groups: (1) phenolic acids and derivatives (20 compounds); (2) flavonoid glycosides and aglycones (12 compounds); (3) iridoid glycosides (13 compounds); and (4) 4 compounds belonging to other classes (Table 1). Data were acquired only in the negative ionization mode of the UHPLC-LTQ OrbiTrap MS instrument; thus, no iridoid aglycones were recorded.

Four hexosyl derivatives of hydroxybenzoic acids (compounds 1, 2, 4, and 5) were identified in the examined samples (Table 1). All other phenolic acid derivatives belonged to hydroxycinnamic acid groups. The MS^2^ base peak of compound **8,** found at retention time 7.03 min and displaying pseudomolecular ion [M−H]^−^ at 369 *m/z*, was identified at 191 *m/z* (deprotonated quinic acid). In this case, the neutral loss was found at 178 *m/z*, which most likely corresponds to the trihydroxycinnamoyl residue. However, the hydroxyl group’s exact position on the benzene nucleus of this compound could not be defined based only on LC/MS ^n^ data. Further fragmentation of 191 *m/z* confirmed the presence of quinic acid. Supplementary Figure S4 presents the proposed structural formula and fragmentation pathway of trihydroxy-cinnamoylquinic acid (**8**). A literature survey revealed that such a compound has not previously been identified in *Nepeta* species. Caffeic acid (13) derivatives are common in *Nepeta* species [17,24]. Caffeoylquinic acid derivatives (3, 9, 10, 15, and 16) and rosmarinic acid (17) were the most abundant phenolic acids in both *Nepeta* species analyzed within the present study. Compound 18, found at Rt = 9.38 min and 313 *m/z*, could be tentatively identified as nepetoidin A or nepetoidin B. Nepetoidin B was previously isolated from root cultures of *Nepeta teydea* [23].

As for flavonoids, compounds from the subgroups of flavonols (compounds 21, 22, 24, 25, and 32) and flavones (compounds 23, 26, 27, 28, 29, 30, and 31) were identified. All of these derivatives, except for compound 24, were previously identified in the genus *Nepeta* [5,17,22]. Compound 24 displayed the molecular ion [M−H]^−^ at 593 *m/z* and MS^2^ base peak fragment at 285 *m/z* by the loss of rhamnosyl and hexosyl group (308 Da). Its MS^2^ fragmentation suggested that the interglycosidic linkage between rhamnose and hexose is of the 1 → 6 type. The relative abundance of the radical molecular ion (284 *m/z*) was not noticed; therefore, this compound was tentatively identified as kaempferol 7-*O*-rutinoside. Three compounds (28, 29, and 32) with the same molecular ions [M−H]^−^ at 313 *m/z*, and displaying very similar fragmentation patterns, were marked as cirsimaritin, ladanein, and kumatakenin, respectively. Tentative identification of these compounds was based on previously published chromatographic and spectral data [22].

The analysis of iridoid glycosides was a great challenge because many isomeric compounds with the same molecular weight could be found. A total of 13 iridoid glycosides have been identified, of which only two (compounds 42 and 43) have so far not been reported in the genus *Nepeta* or in the family Lamiaceae. Compound 42 with retention time 8.25 min and showing molecular ion [M−H]^−^ at 343 *m/z* was tentatively identified as boschnaloside. It gave an MS^2^ base peak at 161 *m/z*, which was generated by the loss of iridoid residue (−182 Da). Further fragmentation of 161 *m/z* ([hexose–H–H_2_O]^−^) was characteristic for sugar cleavage (Supplementary Figure S4), while all other MS^2^ and MS^3^ ions specific for the fragmentation of boschnaloside can be found in the literature [25]. Deoxyloganetic acid pentoside, showing [M−H]^−^ at 329 *m/z* (compound 43), displayed a very similar fragmentation pathway as epideoxyloganic acid (compounds 40 and 41) with pseudomolecular ion [M−H]^−^ at 359 *m/z*. The difference is that compound 43, instead of glucose (hexose), contains pentose in its structure (Supplementary Materials Figure S4). 1,5,9-*e*DLA (compound 41) was previously identified as one of the major iridoid glucosides in various *Nepeta* species, including *N. cataria* [12], *N. cadmea* [13], *N. nuda* subsp. *albiflora* [14], and *N. argolica* [15]. Compound 44 was identified as aucubin.

From the other compounds, the presence of quinic acid (compound 46) and umbelliferone (compound 49) was confirmed using standards. Compound 47, marked as 3,4-dihydroxyphenethyl alcohol 4-*O*-hexoside, was previously detected in *Schizonepeta tenuifolia* (Benth.) Briq. (Lamiaceae) [26], and its fragmentation pathway is depicted in Supplementary Materials Figure S4.

**Figure 3:**
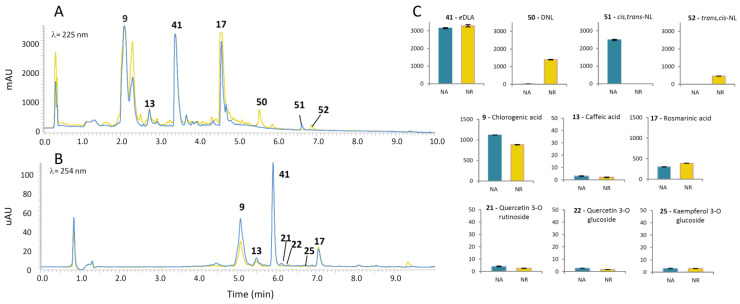
UHPLC/DAD chromatograms of *N. rtanjensis* (yellow line) and *N. argolica subsp. argolica* methanol extracts (blue line) representing: (**A**) major iridoid compounds: *cis,trans*-NL (51) and *trans,cis*-NL (52), both displaying pseudomolecular ion [M+H]^+^ at 167 *m/z*, visible as peaks eluting at Rt = 6.62 min and 6.87 min, respectively; DNL (50), with pseudomolecular ion [M+H]^+^ at 165 *m/z*, eluting at Rt = 5.54 min; 1,5,9-eDLA (41) with [M−H]^−^ at 359 *m/z*, visible as a peak at Rt = 5.54 min; (**B**) major phenolic compounds: 5-*O*-caffeoylquinic acid (9) displaying [M−H]^−^ at 353 *m/z* and eluting at Rt = 5.18 min; caffeic acid (13) with [M−H]^−^ at 179 *m/z* and eluting at Rt = 5.58 min; rosmarinic acid (17) with [M−H]^−^ at 359 *m/z*, visible as a peak eluting at Rt = 7.17 min; quercetin rutinoside (21) with [M−H]^−^ at 609 *m/z,* eluting at Rt = 6.19 min; quercetin 3-*O*-glucoside (22) displaying [M−H]^−^ at 463 *m/z,* eluting at Rt = 6.44 min; kaempferol 3-O-glucoside [M−H]^−^ at 447 *m/z,* eluting at Rt = 6.76 min. Quantitative data (**C**) are presented as µg 100 mg^−1^ FW.

We further adopted two UHPLC/DAD/qqqMS methods to quantify major phenolics and iridoids, respectively. The following iridoid compounds were quantified in methanol extracts of *N. rtanjensis* and *N. argolica* subsp. *argolica*: dehydronepetalactone (DNL, compound 50) *cis,trans*-NL (51), *trans,cis*-NL (52), and 1,5,9-*e*DLA (41). Similarly to our previous studies [17,18,27], the positive ion HESI mode of the UHPLC/DAD/qqqMS instrument was found to be the ionization method of choice for iridoid aglycones (*cis,trans*-NL, *trans,cis*-NL, and DNL), while iridoid glycoside 1,5,9-*e*DLA was analyzed in negative mode. The most abundant iridoid compounds in *N. rtanjensis* sample were 41, 50, and 52, while in *N. argolica* subsp. *argolica* methanol extracts, compounds 41 and 51 predominated (Figure 3A,C). This was in accordance with our previous studies [17,18].

The analysis was targeted towards some of the major phenolic compounds, including phenolic acids (9, 13, 17) and flavonoids (21, 22, 25). The most abundant phenolic compound in methanol extracts of *N. rtanjensis* and *N. argolica* was 5-*O*-caffeoylquinic acid (9) (Figure 3B,C). The second most abundant phenolic compound in both analyzed samples was RA (17) (Figure 3C). Other analyzed phenolic acids and flavonoids were much less abundant in the analyzed samples.

### 2.4. Antimicrobial Activity

Antimicrobial activity of *N. rtanjensis* and *N. argolica* subsp*. argolica* methanol extracts and their major iridoid (*trans,cis*-NL, *cis,trans*-NL, and *e*DLA) and phenolic constituents (RA) were comparatively tested against eight bacterial (*B. cereus, S. aureus, M. flavus, L. monocytogenes, E. faecalis, P. aeruginosa, E. coli* and *S. typhimurium*) and seven fungal food-borne pathogens (*A. fumigatus, A. ochraceus, A. versicolor, A. niger, P. funiculosum, P. ochlochloron* and *P. v. cylopium*) (Table 2). Although two analyzed *Nepeta* species displayed differential qualitative and quantitative content of nepetalactones (Table 1, Figure 3), there was no considerable difference in antimicrobial activity between their methanol extracts. MIC values for *N. rtanjensis* leaf extracts ranged between 0.1 and 0.4 mg mL^−1^, while MIC for *N. argolica* subsp. *argolica* leaf extracts were 0.15–0.3 mg mL^−1^. MBCs of *N. rtanjensis* extracts were 0.2–0.6 mg mL^−1^, while the bactericidal concentrations of *N. argolica* subsp. *argolica* leaf extracts ranged between 0.2 and 0.4 mg mL^−1^. The leaf extract of *N. rtanjensis* exhibited the strongest antibacterial activity against *P. aeruginosa*, *S. aureus*, and *L. monocytogenes*, while *E. coli* was the most sensitive bacterial species to *N. argolica* subsp. *argolica* leaf extract*. E. faecalis* was the most resistant species, with *N. rtanjensis* leaf extract’s inhibitory activity at 0.4 mg mL^−1^ and bactericidal at 0.6 mg mL^−1^, while the MIC value for *N. argolica* subsp. *argolica* extract was 0.3 mg mL^−1^, and MBC was 0.4 mg mL^−1^. The leaf extracts of the two *Nepeta* species also showed great antifungal activity with inhibitory (MIC) activity at 0.05–0.3 mg mL^−1^ and fungicidal (MFC) at 0.1–0.4 mg mL^−1^ (Table 2). The leaf extract of *N. rtanjensis* exhibited stronger antifungal activity against *A. niger* compared to the extract of *N. argolica* subsp. *argolica* and ketoconazole, showing an MIC of 0.05 mg mL^−1^ and MFC of 0.1 mg mL^−1^. The commercial antimycotics possessed powerful antifungal activity with MIC values of 0.0025–0.06 mg mL^−1^ and MFC of 0.005–0.08 mg mL^−1^. Similar antimicrobial results of *N. rtanjensis* methanol extracts were previously reported by Nestorović et al. [4]. This endemic species is well characterized for antimicrobial effects of its EOs and extracts [4,16]. On the other hand, literature dealing with antimicrobial activities of *N. argolica* is rather scarce, and no records on the activity of methanol extracts are reported. *N. argolica* subsp. *dyrphia* essential oil and pure nepetalactones were effective against 25 clinically isolated and commercial strains of *Helicobacter pylori* [3].

**Figure 4:**
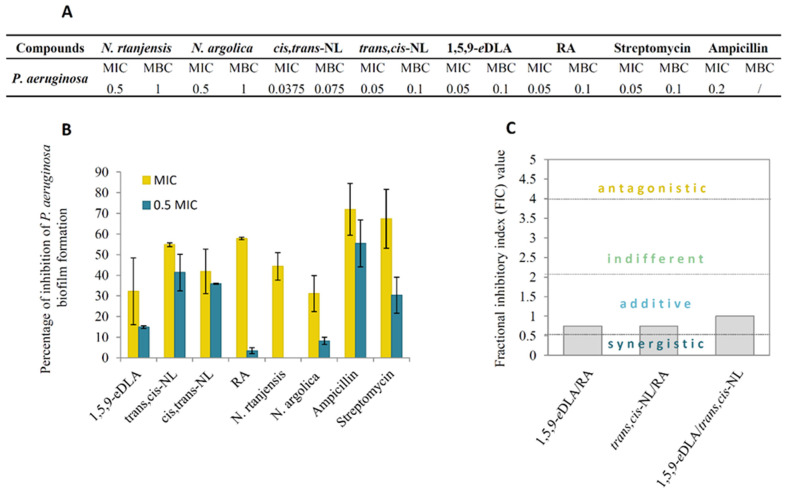
Antibacterial (**A**) and antibiofilm activity (**B**) of *Nepeta rtanjensis* and *N. argolica* subsp. *argolica* methanol extracts, and of *cis,trans*-nepetalactone (*cis,trans*-NL), *trans,cis*-nepetalactone (*trans,cis*-NL), 1,5,9-epideoxyloganic acid (1,5,9-*e*DLA), and rosmarinic acid (RA) against a resistant *P. aeruginosa* strain. Minimal inhibitory (MIC) and lethal concentrations (MBC) were determined, and streptomycin and ampicillin were adopted as reference compounds. (**C**) Antibacterial interaction against *P. aeruginosa* of the following compound combinations: *trans,cis*-NL/1,5,9-*e*DLA, *trans,cis*-NL/RA, and 1,5,9-*e*DLA/RA.

The formation of biofilm is the initial step that leads to the overgrowth of food-borne bacteria, and in the food industry biofilms create a persistent contamination source. The examined *Nepeta* sp. extracts/compounds have shown significant potential in inhibiting the growth of a resistant strain of bacterial pathogen *P. aeruginosa* (Figure 4A). This opportunistic pathogen is used as a model system for biofilm formation due to its ability to establish biofilms on different surfaces and because it is frequently present in water, dairy products, meat, and food of plant origin [28]. Pure compounds have shown more promising activity than extracts, with MIC values of 0.0375–0.05 mg mL^−1^ and MBC values of 0.075–0.1 mg mL^−1^.

Among tested extracts and pure compounds, the most substantial antibiofilm potential could be observed for *trans,cis*-NL (Figure 4B). The application of this compound in a concentration equal to previously determined MIC caused 54.8% inhibition while one-half of the MIC reduced biofilm biomass formation by 41.4%. The other two iridoid compounds, *cis,trans*-NL and 1,5,9-*e*DLA, also displayed significant antibiofilm potential, inducing around 41.8% and 32.2% inhibition when applied with MICs, respectively. The efficiency of RA, which induced 57.8% inhibition with MIC, was drastically reduced to 3.4% with one-half MIC. Likewise, antibiofilm activity of *N. nuda* tincture, rich in *e*DLA, has been demonstrated against *E. faecalis* biofilm formation [29].

To determine potential antimicrobial interactions of the major compounds found in *N. rtanjensis* leaf extracts against *P. aeruginosa*, three combinations of compounds (RA+1,5,9-*e*DLA; RA+*trans,cis-*NL, and 1,5,9-*e*DLA+*trans,cis-*NL) were examined. The results have shown that all three compound combinations have an additive effect on *P. aeruginosa* growth inhibition (Figure 4C), which might, at least partially, be responsible for the pronounced antimicrobial activity of *N. rtanjensis* methanol extracts. Taking into account the recorded iridoid and phenolic profiles in methanol extracts (Table 1) and the known antimicrobial activities for some of the compounds which are available in the literature [1,5], the contribution of minor constituents of methanol extracts to the overall antimicrobial activity should not be neglected.

### 2.5. Immunomodulating Activity

**Figure 5:**
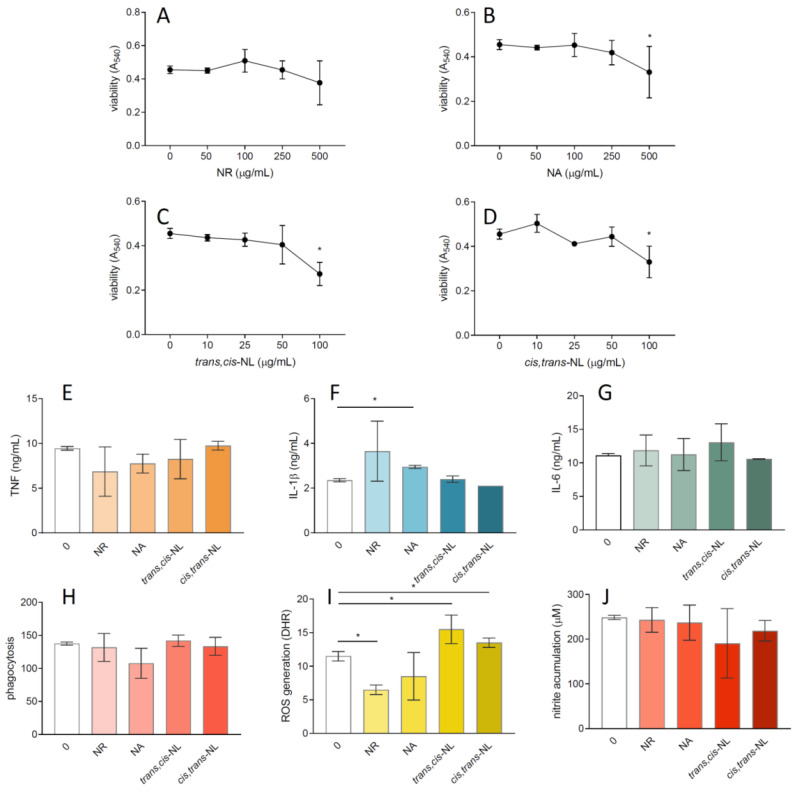
Effects of *N. rtanjensis* and *N. argolica* subsp. *argolica* methanol extracts and nepetalactones (*cis,trans*-NL and *trans,cis*-NL) on macrophages. Macrophages were stimulated with LPS and treated with various concentrations of extracts and nepetalactones for 24 h. Subsequently, cell viability was determined by CV test (**A**–**D**), while productions of cytokines (**E**–**G**) and nitrite (**J**) were measured in the culture supernatants. Phagocytosis (**H**) and ROS levels (**I**) in the absence (0) or presence of extracts or nepetalactones (250 or 50 µg mL^−1^) were determined by cytofluorimetry. Data are presented as mean + SD from repeated experiments. * *p* < 0.05 is statistically significant.

The intensity of the inflammatory response induced by food-borne infections could be responsible for the severity of the tissue lesions; therefore, we have also tested the influence of *N. rtanjensis* and *N. argolica* subsp. *argolica* extracts and pure nepetalactones (*cis,trans*-NL and *trans,cis*-NL) on macrophages’ activity. After 24 h of cultivation, the viability of macrophages was reduced under the influence of *N. rtanjensis* and *N. argolica* subsp. *argolica* leaf extracts (Figure 5A,B) at concentrations of 500 µg mL^−1^ and NLs at concentrations of 100 µg mL^−1^ and higher (Figure 5C,D). Therefore, extracts in concentrations of 250 µg mL^−1^ and NLs in concentrations of 50 µg mL^−1^ were used for in vitro experiments with macrophages. Both extracts and NLs exhibited no significant effect on nitrite (Figure 5J), TNF (Figure 5E), or IL-6 production (Figure 5G). Additionally, the level of phagocytosis per macrophage cell remains unchanged after treatment with the analyzed extracts and NLs (Figure 5H). Both *N. rtanjensis* and *N. argolica subsp. argolica* leaf extracts induced the elevation of proinflammatory cytokine IL-1*β*, while all tested compounds showed no significant effects. ROS production per cell was significantly reduced after treatment with *N. rtanjensis* leaf extract, while the same concentration of *N. argolica* subsp. *argolica* had no effect (Figure 5I). Conversely, treatment with 50 µg mL^−1^ *trans,cis*-NL and *cis,trans*-NL increased the ROS production in macrophages. 

A previous study has demonstrated the strong anti-inflammatory effect of *N. argolica* subsp. *argolica* extracts [24]. It has been shown that the extracts of *N. dschuparensis*, rich in NL, significantly reduce levels of proinflammatory cytokine IL-1*β* and cyclooxygenase 2 (COX-2) in a cerebral ischemia-reperfusion model [30]. Similarly, *n*-butanol extract from *N. asterotricha* has decreased the production of IL1-*β*, IL-6, TNF and ROS in J774A.1 cell line of macrophages [21]. In contrast to earlier findings, results of the present study indicate that nepetalactones are probably minor contributors to the overall immunomodulatory effects in the tested culture of macrophages and two *Nepeta* species.

### 2.6. In Conclusion

The methanol extracts of *N. argolica* subsp*. argolica*, and especially *N. rtanjensis*, show very promising antibacterial and antifungal potential, owing to the high content of iridoid and phenolic compounds and their additive interactions. Iridoids (*trans,cis*-NL, *cis,trans*-NL, and 1,5,9-*e*DLA) and dominant phenolic constituents (RA) of *Nepeta* species may be recommended individually or in mixtures to preserve foods and beverages as more “green/organic” alternatives for the extension of the product shelf life. Furthermore, they can be utilized to prevent and treat diseases caused by common and emerging food-borne pathogens such as *L. monocytogenes*, *E. coli*, *S. aureus, P. aeruginosa*, *Penicillium* sp. and *Aspergillus* spp., as well as for pathological conditions to reduce or stimulate the occurrence of proinflammatory responses. The results of the present study support the hypothesis that nepetalactone stereochemistry determines the antimicrobial activity of *Nepeta* species, and that iridoid glucoside 1,5,9-*e*DLA is an equally potent antimicrobial as nepetalactones. Taking into account their well-known health benefits for humans, iridoids of *Nepeta* sp. are prospective functional food additives, especially 1,5,9-*e*DLA, which is not volatile and is more stable in food formulations.

## 3. Materials and Methods

### 3.1. Chemicals and Reagents

Solvents for HPLC/MS analyses (acetonitrile, acetic and formic acids) were of LC/MS grade, obtained from Fisher Scientific (Loughborough, UK). Methanol for plant extraction (99.8%, HPLC grade) and semi-preparative (SP) HPLC/DAD analysis was HPLC grade (AppliChem GmbH, Darmstadt, Germany). Ultra-pure deionized water was generated using the Water Purification System (New Human Power I Integrate, Human Corporation, Republic of Korea). Standards of phenolics were purchased from Sigma-Aldrich (Steinheim, Germany).

### 3.2. Isolation of cis,trans- and trans,cis-Nepetalactone

#### 3.2.1. Origin of Essential Oils

Essential oil (EO) of dry *Nepeta rtanjensis* Diklić & Milojević flowering plants, containing 72% of *trans,cis*-NL, and 16% of *cis,trans*-NL, was obtained by hydrodistillation in a Clevenger-type apparatus, as previously described by Skorić et al. [31]. The EO of *Nepeta cataria* L., containing 90% of *cis,trans*-NL, was provided by Dr. Michael Birkett from Rothamsted Research (Harpenden, UK).

#### 3.2.2. Semi-Preparative HPLC/DAD Fractionation and Isolation of *cis,trans*- and *trans,cis*-Nepetalactone

Semi-preparative HPLC fractionation of EOs, diluted in 99.6% methanol (w:v = 1:50), was performed on an HPLC system, model HP1100 with DAD (Hewlett Packard, Santa Clara, CA, USA), connected to a Fraction collector 1200 Series (Agilent Technologies, Waldbronn, Germany). Chromatographic separation was performed using a ZORBAX SB-C18 (9.4 × 100 mm, 5 µm) column (Agilent Technologies, Waldbronn, Germany), thermostated at 40 °C. The mobile phase consisted of (A) deionized water and (B) methanol, which were applied in the following gradient elutions: 80–10% A for 30 min; 10% A during the next 10 min; 10–80% A in the next 5 min. The flow rate was set to 2.000 mL min^−1^, and the detection wavelength was λ = 230 nm. The injection volume was 100 µL. Collected fractions of *cis,trans-* and *trans,cis-*NL (containing the desired compounds in ~69% and 70% methanol, respectively) were mixed with hexane (v:v = 3:2), vigorously vortexed for 2 min, and the upper phase containing *cis,trans*- or *trans,cis*-NL in hexane was transferred into glass vials. Solvent was evaporated at room temperature overnight, and samples were additionally dried under the N gas flow.

#### 3.2.3. GC-MS Identification of Nepetalactone Diastereoisomers in Isolated Fractions

The analysis was performed using GCMS QP2010 plus, equipped with an AOC 5000 injector (Shimadzu, Kyoto, Japan), and ZB-1 column (Phenomenex, L = 30 m, ID = 0.25 mm, df = 0.50 µm). Samples (1 µL) were injected in the split mode (1:30), with injector temperature set to 250 °C. Mass spectra were acquired in EI mode (±70 eV) in the *m/z* range 40–400 amu (SCAN) mode together with Single Ion Monitoring (SIM) mode, and helium (He, 99.999%) was used as a carrier gas with a flow rate of 1 mL min^−1^. The column was heated linearly from 40 °C to 280 °C with a gradient of 6 °C min^−1^ and held at 280 °C for 5 min. Ion source temperature was set to 280 °C; interface temperature to 290 °C; SCAN 40-400 *m/z*; SIM 164, 166. Identification of constituents was performed by comparing their mass spectra to those from Wiley8, NIST05, and FFNSC3 libraries, using different search engines.

#### 3.2.4. UHPLC-MS^2^ Analysis of Isolated Fractions

Analyses were performed using a Dionex Ultimate 3000 UHPLC system (ThermoFisher Scientific, Bremen, Germany) equipped with a diode array detector (DAD) and a TSQ Quantum Access Max triple-quadrupole (QQQ) mass spectrometer (ThermoFisher Scientific, Basel, Switzerland). Chromatographic separation of NLs was performed on a Hypersil gold C18-column (50 × 2.1 mm) with 1.9 μm particle size (ThermoFisher Scientific, Fair Lawn, NJ, USA), thermostated at 40 °C. The mobile phase consisted of (A) 0.2% formic acid in water and (B) acetonitrile (MS grade, Fisher Scientific, Loughborough, UK), which were applied at the flow rate and gradient elution, as previously described by Mišić et al. [17]. Settings of the TSQ Quantum Access Max QQQ mass spectrometer were as previously described [18]. The MS data were acquired in positive ionization mode, and the collision-induced fragmentation, in a selected reaction monitoring (SRM) experiment, was performed using argon as the collision gas, with collision energy (cE) set to 30 eV.

#### 3.2.5. NMR Analysis for the Structural Characterization of Isolated *cis,trans*- and *trans,cis*-Nepetalactone

Samples were dissolved in chloroform-*d* and further used in NMR analysis. The NMR spectra were recorded on a Bruker AVANC 500 NMR spectrometer equipped with a BBI 5 mm probe head at 298 K. Samples were dissolved in 99.8 chloroform-*d* (SigmaAldrich, USA) with 0.03% (v/v) TMS. Recorded spectra were processed by MestReNova (v14.0.0–23239, Mestrelab Research S. L.).

*trans,cis*-Nepetalactone: ^13^C NMR (125 MHz, chloroform-*d*): *δ* 170.4 (C-1), 136.1 (C-3), 120.6 (C-4), 49.3 (C-7a), 37.8 (C-7), 32.3 (C-6), 30.2 (C-4a), 26.4 (C-5), 17.7 (C-9), 14.5 (C-8); ^1^H NMR (500 MHz, chloroform -*d*): *δ* 6.24 (m, H-3), 2.70 (m, H-7α), 2.51 (m, H-4aα), 2.34 (dd, *J* = 15.3, 6.7 Hz, H-7aβ), 2.14 (m, H-6α), 1.96 (m, H-5β), 1.71 (dd, *J* = 1.5, 1.4 Hz, H-8), 1.40 (m, H-6β), 1.38 (m, H-5α), 1.15 (d, *J* = 7.0, Hz, H-9).

*cis,trans*-Nepetalactone: ^13^C NMR (125 MHz, chloroform-*d*): *δ* 171.1 (C-1), 133.8 (C-3), 115.5 (C-4), 49.6 (C-7a), 41.0 (C-4a), 40.0 (C-7), 33.3 (C-6), 31.2 (C-5), 20.5 (C-9), 15.6 (C-8); ^1^H NMR (500 MHz, chloroform -*d*): *δ* 6.17 (bs, H-3), 2.75 (m, H-4aα), 2.45 (dd, *J* = 9, 8.5 Hz, H-7aα), 2.38 (m, H-7α), 2.03; 1.56 (m, H-5), 1.91; 1.23 (m, H-6), 1.62 (m, H-8), 1.20 (d, *J* = 6.7 Hz, H-9).

### 3.3. Isolation of 1,5,9-Epideoxyloganic Acid

#### 3.3.1. Plant Material

Above-ground parts were harvested in 2019, from *Nepeta rtanjensis* Diklić & Milojević plants grown in the greenhouse of the Institute for Biological Research “Siniša Stanković”—National Institute of Republic of Serbia, University of Belgrade (IBISS-UB), Serbia.

#### 3.3.2. Preparation of Methanol Extracts

Plant material (110 g FW) was ground in liquid nitrogen (LN) and diluted in 250 mL 96% methanol. Extraction was performed overnight at 4 °C. The next day, extraction was continued in an ultrasonic bath at room temperature for 15 min. Following filtration, the sample was evaporated in a vacuum evaporator (Rotavapor RII, Buchi, Switzerland) and additionally dried under a N gas flow. Samples (4.5 g DE) were stored at 4 °C until use.

#### 3.3.3. HRMS Analysis of *N. rtanjensis* Methanol Extract

Dry methanol extract of *N. rtanjensis* was diluted in 96% methanol (1:1 = w:v) in an ultrasonic bath at 100 Hz and 25 °C, and filtered through 13 mm (pore size 0.22 µm) filters (ANPEL Laboratory Technologies (Shanghai) Inc., Shanghai, China). The sample was chromatographically separated on a Waters Acquity UPLC Q-TOF system using an Acquity UPLC ^®^ BEH C18 column (particle size 1.7 µm, dimensions 2.1 × 100 mm). The mobile phase consisted of 0.1% formic acid in water (A) and 0.1% formic acid in acetonitrile (B). The mobile phase was eluted with a flow rate of 0.3 mL min^−1^, and the elution gradient was as follows: 0–1 min 5% B, 1–12 min 5–95% B, 12–12.2 min 95–5% B, 12.2–15 min 5% B. The LC system was connected to the mass spectrometer with an electrospray ionization (ESI) source operating in negative ionization mode, with the following operating conditions: mass range of 50 Da to 1200 Da, low collision energy (cE) of 10 keV, high cE at 20 keV, target column temperature of 45 °C, target sample temperature of 15 °C, resolution of 20,000, source desolvation temperature of 350 °C, source temperature of 120 °C, capillary voltage of 2.50 kV, cone gas flow of 50.0 l h^−1^, desolvation gas flow of 750.0 l h^−1^, nebulizer gas flow of 6.5 bar, trap gas flow of 2.00 mL min^−1^, and IMS gas flow of 90.00 mL min^−1^.

#### 3.3.4. Fractionation of *N. rtanjensis* Methanol Extract

The sample was gradually homogenized in ODS (w:w = 1:2). Fractionation was performed using a Soochow Medium Pressure System Easystep TM 1010 (Soochow High Tech Chromatography Co. Ltd., Suzhou, China), connected to a fraction collector SmartCell-3060 (Soochow High Tech Chromatography Co. Ltd., Suzhou, China). The extract was chromatographically separated on an ODS column (49 mm (I.D.) × 460 mm (L), (Soochow High Tech Chromatography CO, LTD) and eluted with methanol and water in a stepwise manner (30%, 40%, 50%, 60%, 70%, 80%, 100%), with a flow rate of 100 mL min^−1^. Collected fractions (119 in total) were filtered through nitrocellulose filters (2 µm) and checked for the content of analytes. Analytical HPLC and ESIMS spectra were recorded on a Waters 2695 instrument with a 2998 PAD coupled with a Waters Acquity ELSD and a Waters 3100 SQDMS detector. Fractions containing targeted 1,5,9-*e*DLA, which displayed λ_max_ at 236 nm and pseudomolecular ion [M+H]^+^ at *m/z* 359, were pooled and vacuum-evaporated at 40 °C.

#### 3.3.5. Preparative HPLC/DAD Isolation of 1,5,9-Epideoxyloganic Acid

Dry 1,5,9-*e*DLA acid fraction was diluted in methanol (10 mg mL^−1^), and 500 µL of the solution was repetitively injected onto the preparative HPLC system: Waters 2545 Binary Gradient Module instrument with a Waters 2489 UV/Visible Detector. The sample was chromatographically separated using a Waters Sunfire Prep C18 OBD ^TM^ column (5 μm, 30 × 150 mm) at room temperature. Fractions were collected using Waters 2767 Sample Manager. Elution was performed using (A) 0.1% formic acid in water and (B) 0.1% formic acid in ACN as mobile phases, at the flow rate of 30 mL min^−1^, and elution gradient was 10–40% ACN in 40 min. The wavelength of the UV/Vis detector was set to 236 nm. Fractions containing 1,5,9-*e*DLA were pooled, and solvents were vacuum-evaporated.

#### 3.3.6. NMR Structure Confirmation of 1,5,9-Epideoxyloganic Acid

NMR data were recorded on a BrukerAvance III-500 spectrometer and processed by MestReNova (v14.0.0-23239, Mestrelab Research S. L.).

Results for 1,5,9-Epideoxyloganic acid: ^13^C NMR (125 MHz, Methanol-*d4*): *δ* 152.9 (C-3), 113.9 (C-4), 104.0 (C-1′), 100.7 (C-1), 78.4 (C-5′), 78.2 (C-3′), 75.3 (C-2′), 71.2 (C-4′), 62.6 (C-6′), 44.4 (C-9), 37.2 (C-8), 34.2 (C-5), 33.7 (C-7), 32.4 (C-6), 16.7 (C-10); ^1^H NMR (500 MHz, Methanol-*d*_4_): *δ* 7.35 (s, H-3), 5.27 (d, *J* = 4.0 Hz, H-1), 4.54 (d, *J* = 7.9 Hz, H-1′), 3.80 (dd, *J* = 12.0, 2.0 Hz, H-6′β), 3.65 (dd, *J* = 12.0, 4.6 Hz, H-6′α), 3.20-3.40 (m, H-2′, 3′, 4′, overlapped with the signals due to the residual of methanol), 3.19 (dd, *J* = 7.9, 8.3 Hz, H-2′), 2.88 (ddd, *J* = 8.4, 5.0, 5.0 Hz, H-5), 2.37 (td, *J* = 8.5, 4.0 Hz, H-9), 2.26 (m, H-8), 1.97 (m, H-6α), 1.74 (m, H-7α), 1.59 (m, H-6β), 1.27 (m, H-7β), 1.04 (d, *J* = 7.2 Hz, 10-Me).

### 3.4. Phytochemical Characterization of Methanol Extracts of Nepeta rtanjensis and N. argolica subsp. argolica Grown In Vitro

#### 3.4.1. Plant Material

In vitro cultures of *Nepeta rtanjensis* Diklić & Milojević and *N. argolica* Bory & Chaub. subsp. *argolica* (syn. *N. sibthorpii* Bentham, *N. argolica* Bory & Chaub., according to The Plant List and Euro+Med Plant Base) were established from seeds as previously described by Mišić et al. [17] and Aničić et al. [18]. Plant material for the experiment was obtained by the micropropagation of plants using single-node stem segments as explants, as previously described [17].

#### 3.4.2. Preparation of Methanol Extracts

Above-ground parts of *N. rtanjensis* and *N. argolica subsp. argolica* cultured in vitro were harvested, weighed, and ground in LN. The material was extracted with 99.8% methanol (w:v = 1:10) by vortexing for 1 min and in an ultrasonic bath for 15 min. After centrifugation for 20 min at 10,000× *g*, the supernatants were filtered through 0.2 μm cellulose filters (Agilent Technologies, Santa Clara, CA, USA), and stored at 4 °C until use.

#### 3.4.3. Identification of Phytochemicals in Methanol Extracts Using UHPLC-LTQ OrbiTrap XL

UHPLC separation was carried out on an Accela 600 system coupled to the LTQ OrbiTrap XL mass spectrometer (ThermoFisher Scientific, Bremen, Germany). The analytical column used for separation was a Syncronis C18 column (100 × 2.1 mm, 1.7 µm) heated to 40 °C. The mobile phase was composed of (A) 0.1% formic acid (MS grade, Sigma-Aldrich, Steinheim, Germany) and (B) acetonitrile (MS grade, Merck, Darmstadt, Germany) with 0.1% formic acid. The flow rate was set to 0.175 mL min^−1^ and the gradient elution program was previously described by Banjanac et al. [32]. MS parameters of the ion source and the other MS settings were as previously described in Banjanac et al. [32].

#### 3.4.4. UHPLC/qqqMS^2^ Quantification of Major Iridoids in Methanol Extracts

Quantification of iridoids in *N. rtanjensis* samples was performed using the UHPLC/qqqMS2 instrument settings described in Section 3.2.4. The MS data were acquired simultaneously in positive (*cis,trans*- and *trans,cis*-NL) and negative mode (1,5,9-*e*DLA) in selected reaction monitoring (SRM) mass spectrometric scanning mode, with collision-induced fragmentations performed using argon as the collision gas, and collision energy (cE) set to 30 eV. An SRM experiment for quantitative analysis was performed using two diagnostic MS^2^ fragments for each compound. Quantifications of *cis,trans*-NL, *trans,cis*-NL, and 1,5,9-*e*DLA acid were performed using calibration curves of isolated standards, and the total amount of compounds in samples was expressed as μg 100 mg^−1^ dry extract.

#### 3.4.5. UHPLC/qqqMS^2^ Quantification of Major Phenolics in Methanol Extracts

Phenolics in the methanol extract of *N. rtanjensis* were chromatographically separated on a Syncronis C18 column (100 × 2.1 mm) with 1.7 μm particle size (ThermoFisher Scientific, USA), thermostated at 40 °C. The mobile phase consisted of (A) 0.1% acetic acid in water and (B) acetonitrile (MS grade, FisherScientific, UK), which were applied in the following gradient elutions: 5% B in the first minute, 5–95% B from 1.0 to 16.0 min, from 95% to 5% B for 16.0–16.2 min, and 5% B until the 20th min. The flow rate was set to 0.3 mL min^−1^, and the injection volume was 5 μL. Settings of the TSQ Quantum Access Max QQQ mass spectrometer for the time-selected reaction monitoring (tSRM) experiment were as previously described in Čolić et al. [33]. Xcalibur software (version 2.2) was used for instrument control, data acquisition, and analysis. The total amount of compounds in samples was calculated based on the calibration curve of pure compounds and expressed as μg 100 mg^−1^ dry extract.

### 3.5. Antimicrobial Activity of Two Nepeta Species Methanol Extracts and Their Major Iridoids

#### 3.5.1. Microorganisms and Culture Conditions

For the bioassays, eight bacteria species were used, including three Gram-negative: *Escherichia coli* (ATCC 35210), *Pseudomonas aeruginosa* (ATCC 27853), *Salmonella* Typhimurium (ATCC 13311), and five Gram-positive bacteria: *Listeria monocytogenes* (NCTC 7973), *Bacillus cereus* (human isolate), *Enterococcus faecalis* (ATCC 19433), *Micrococcus flavus* (ATCC 10240), and *Staphylococcus aureus* (ATCC 6538). A resistant strain of *Pseudomonas aeruginosa*, used to determine antibiofilm activity and for the assessment of synergism between major constituent of methanol extracts, was obtained as described in Kartsev et al. [34].

Seven fungal species were also used: *Aspergillus ochraceus* (ATCC 12066), *A. niger* (ATCC 6275), *A. fumigatus* (ATCC 9197), *A. verrucosum* (ATCC 11730), *Penicillium funiculosum* (ATCC 36839), *P. ochrochloron* (ATCC 9112), and *P.v. cyclopium* (food isolate). All of the organisms tested were from the Mycological Laboratory, Department of Plant Physiology, IBISS-UB. The micromycetes were maintained on malt agar (MA), and bacteria on Mueller–Hinton agar medium (MH). Cultures were stored at 4 °C and sub-cultured once per month.

#### 3.5.2. Microdilution Method

Antimicrobial activity was analyzed by adopting the modified microdilution technique as previously described by Soković et al. [35].

Determination of minimum inhibitory concentrations (MICs), and the minimum bactericidal concentrations (MBCs) and fungicidal concentrations (MFCs) were performed by a serial dilution technique using 96-well microtiter plates. The compounds investigated were dissolved in 5% DMSO in concentration 2 mg mL^−1^, and for the extracts 10 mg mL^−1^, and added in broth medium with inoculum. 

Streptomycin and commercial fungicide ketoconazole were used as positive controls (0.1–2 mg mL^−1^).

#### 3.5.3. Antibiofilm Activity

The method was performed as described in Sirakanyan et al. [36]. Briefly, *P. aeruginosa* resistant strain was incubated with MIC and subMIC (0.5 MIC) concentrations of tested compounds or methanol extracts in Tryptic soy broth (TSB) enriched with 2% glucose at 37 °C. After 24 h, each well was washed twice with sterile PBS (phosphate-buffered saline, pH 7.4) and fixed with methanol for 10 min. After the removal of methanol, the plate was air-dried. Biofilm was stained with 0.1% crystal violet (Bio-Merieux, France) for 30 min. Wells were washed with water, air-dried, and 100 μL of 96% ethanol (Zorka, Serbia) was added. The absorbance was read at 620 nm on a Multiskan™ FC Microplate Photometer, Thermo Scientific ™. The percentage of inhibition of biofilm formation was calculated according to the following formula:

[(A_620_ control–A_620_ sample)/A_620_ control] × 100.

#### 3.5.4. Antimicrobial Interaction between *trans,cis*-NL, 1,5,9-eDLA, and RA

This method was carried out using 96-well microplates containing TSB medium for resistant *P. aeruginosa* strain, supplemented with *trans*,*cis*-NL, 1,5,9-*e*DLA, and RA in concentrations ranging from 1/16 to 4 × MIC. Compounds were combined on the microplate in a checkerboard style, as described in Nikolić et al. [37]. The microplates were incubated for 24 h at 37 °C. Three combinations (*trans*,*cis*-NL x 1,5,9-*e*DLA; *trans*,*cis*-NL × RA; 1,5,9-*e*DLA × RA) were used to assess the potential combinatory antimicrobial activity of targeted compounds. The MIC of compound combinations was determined as described for the antimicrobial assay. The fractional inhibitory concentration index (FICI) was calculated using the following formula:

FICI = FIC1^0^*/*MIC1^0^ + FIC2^0^/MIC2^0^

FIC1^0^ and FIC2^0^ are the MICs of compounds combined, and MIC1^0^ and MIC2^0^ represent the MIC values of individual compounds. The results were interpreted by the following cut-offs: FIC ≤ 0.5 synergistic, >0.5 < 2 additive, ≥2 < 4 indifferent, and FIC > 4 antagonistic effects.

### 3.6. Immunomodulating Activity of Two Nepeta Species Methanol Extracts and Isolated Nepetalactones

#### 3.6.1. Cells and Cell Cultures

Resident peritoneal cells were isolated from Dark Agouti rats that were maintained in the animal facility of the IBISS-UB. The experimental procedures were approved by the local Ethics Committee (IBISS-UB, No (02–09/16)). Macrophages were isolated as previously described [38]. Following isolation, macrophages were stimulated with 10 ng mL^−1^ lipopolysaccharide (LPS, Sigma-Aldrich) and treated with extracts and NLs (*cis,trans*-NL and *trans,cis*-NL).

#### 3.6.2. Cell Viability Assays

A Crystal Violet (CV) test was used to determine the number of living, adherent macrophages. At the end of the treatment, supernatants were removed; cells were washed with PBS and fixed with methanol. The cells were stained with 0.1% CV solution. After staining, plates were washed with water and dye was dissolved in 33% acetic acid solution. The absorbance of the solution was measured in triplicates by an automated microplate reader (LKB 5060-006, LKB, Vienna, Austria) at 540 nm. The intensity of the dye corresponded to the viability of the cells.

#### 3.6.3. ELISA Test for Determination of Cytokines

Cytokine concentration in cell culture supernatants was determined using the sandwich ELISA assay. For this purpose, 96-well MaxiSorp microtiter plates (Nunc, Roskilde, Denmark) and commercial antibody pairs were used to measure the concentrations of the respective cytokines: IL-1*β*, IL-6, TNF (eBioscience). The process was performed according to the protocols provided by the manufacturer. The lower limit of detection was 30 pg mL^−1^, while the upper limit of detection was 10 ng mL^−1^ for all of the ELISA tests performed. The concentrations of cytokines (ng mL^−1^) were determined using the standard curves made with appropriate concentrations of the recombinant cytokines.

#### 3.6.4. Detection of Phagocytosis

The level of phagocytosis in macrophages was determined by flow cytofluorimetry. Fluorescently labeled latex beads (1 µm, yellow green, Sigma-Aldrich) were preopsonized in PBS with 50% FCS, 1 h at 37 °C. A preopsonized bead solution was added over macrophages (1 × 105/well) and incubated at 37 °C for an additional hour. Following this, the solution was removed, the cells were washed twice with PBS, stripped off, and analyzed by flow cytofluorimetry. The results are presented as the mean fluorescence intensity (mfi) of the population.

#### 3.6.5. Detection of ROS and NO Production

ROS generation was assessed by dihydrorhodamine 123 (DHR, SigmaAldrich, Steinheim, Germany) staining. The cells were pretreated with extracts and nepetalactones for 24 h, then incubated in the presence of 1 µM DHR for 30 min, stimulated with LPS for an additional 90 min, and analyzed by flow cytofluorimetry. The results are presented as mean fluorescence intensity (mfi) of the population. The production of NO in the supernatants of treated macrophages was determined by a Griess reaction based on nitrite accumulation. Briefly, triplicate aliquots of cell-free supernatants were mixed with an equal volume of Griess reagent (1:1 mixture of 0.1% naphthylethylenediaminedihydrochloride and 1% sulphanilamide in 5% H_3_PO_4_). The absorbance of the solutions was measured by microplate reader at 540 nm, and the nitrite concentration was calculated based on the standard curve for NaNO_2_.

## Figures and Tables

**Table 1:** Orbitrap-MS ^n^ analysis of phenolics and iridoid glucosides in methanol extracts of *N. rtanjensis* (NR) and *N. argolica* subsp. *argolica* (NA).

No.	Compound Name	*T_R_*, Min	Molecular Formula, [M–H]^−^	Calculated Mass, [M–H]^−^	Exact Mass, [M–H]^−^	Δ mDa	MS^2^ Fragments, (% Base Peak)	MS^3^ Fragments, (% Base Peak)	MS^4^ Fragments, (% Base Peak)	NA	NR
***Phenolic Acid Derivatives***											
**1**	**DihyDroxybenZoic Acid Hexoside**	5.76	C_13_H_15_O_9_^−^	315.07216	315.06858	3.58	108 (8), 109 (11), 152 (41), **153** (100), 163 (8), 165 (14), 269 (7)	**109** (100)	ND	+	+
**2**	**Syringic Acid Hexoside**	6.17	C_15_H_19_O_10_^−^	359.09837	359.09405	4.32	182 (4), **197** (100), 198 (8), 313 (7)	138 (8), 153 (18), **182** (100)	137 (3), 138 (4), **167** (100)	+	–
**3**	**3-*O*-CafeOylquinic Acid**	6.38	C_16_H_17_O_9_^−^	353.08781	353.08319	4.62	135 (8), 179 (37), **191** (100), 192 (4)	**85** (100), 93 (52), 111 (32), 127 (91), 171 (21), 173 (59)	**57** (100)	+	+
**4**	**DihyDroxybenZoic Acid Hexoside Isomer**	6.57	C_13_H_15_O_9_^−^	315.07216	315.06872	3.44	109 (5), 135 (4), **153** (100), 154 (7)	109 (50), **135** (100)	**91** (100)	+	+
**5**	**HydroxybenZoic Acid Hexoside**	6.80	C_13_H_15_O_8_^−^	299.07724	299.07395	3.29	93(3), **137** (100)	**93** (100)	ND	+	+
**6**	**Caffeic Acid Hexoside**	6.88	C_15_H_17_O_9_^−^	341.08781	341.08369	4.12	135 (10), **179** (100), 180 (9), 181 (3), 251 (6), 281 (8), 293 (3)	**135** (100)	ND	+	+
**7**	**Sinapic Acid *^a^***	6.93	C_11_H_11_O_5_^−^	223.06120	223.05872	2.48	164 (22), 165 (3), 177 (12), 179 (39), 180 (6), **208** (100), 209 (11)	135 (4), 149 (16), 163 (10), **164** (100), 193(8)	134 (5), 135 (42), **149** (100)	+	+
**8**	**TrihyDroxy-CinnamoylQuinic Acid**	7.03	C_16_H_17_O_10_^−^	369.08272	369.07675	5.97	149 (14), 173 (62), **191** (100), 193 (28), 194 (23), 195 (23), 245 (36)	**85** (100), 93 (71), 109 (29), 111 (29), 127 (92), 173 (64)	ND	+	+
**9**	**5-*O*-Café Oylquinic Acid *^a^***	7.04	C_16_H_17_O_9_^−^	353.08781	353.08303	4.78	179 (9), **191** (100)	**85** (100), 93 (56), 109 (23), 111 (34), 127 (91), 173 (57)	57 (100)	+	+
**10**	**5-*O*-CafeOylquinic Acid Isomer**	7.49	C_16_H_17_O_9_^−^	353.08781	353.08321	4.60	179 (7), **191** (100)	**85** (100), 93 (51), 111 (29), 127 (84), 171 (25), 173 (54)	**57** (100)	+	+
**11**	**Danshensuan C**	7.53	C_18_H_17_O_9_^−^	377.08781	377.08261	5.20	**359** (100), 360 (3)	133 (5), **161** (100), 179 (19), 197 (21), 223 (8)	**133** (100)	–	+
**12**	**5-*O*-CaffeOylshikimic Acid**	7.74	C_16_H_15_O_8_^−^	335.07724	335.07371	3.53	135 (24), 161 (4), **179** (100), 180 (7)	**135** (100)	ND	+	+
**13**	**Caffeic Acid *^a^***	7.78	C_9_H_7_O_4_^−^	179.03498	179.03311	1.87	**135** (100), 136 (14), 161 (7)	79 (37), 107 (80), **117** (100)	ND	+	+
**14**	**Feruloylquinic Acid**	8.01	C_17_H_19_O_9_^−^	367.10346	367.09822	5.24	173 (5), **191** (100), 192 (7), 193 (4)	**85** (100), 93 (49), 109 (29), 111 (41), 127 (78), 173 (66)	ND	+	–
**15**	**DicaffeoyLquinic Acid**	8.67	C_25_H_23_O_12_^−^	515.11950	515.11446	5.04	173 (18), 179 (12), 191 (7), 203 (7), 335 (17), **353** (100), 354 (14)	135 (13), **173** (100), 179 (73), 191 (46)	59 (10), **93** (100), 111 (66), 127 (13), 155 (28)	+	–
**16**	**Dicaffeoylquinic acid isomer**	9.07	C_25_H_23_O_12_^−^	515.11950	515.11437	5.13	173 (8), 179 (6), 203 (15), 255 (6), 299 (10), **353** (100), 354 (15)	135 (11), **173** (100), 179 (66), 191 (38)	71 (15), **93** (100), 109 (10), 111 (44), 155 (18)	–	+
**17**	**Rosmarinic acid *^a^***	9.33	C_18_H_15_O_8_^−^	359.07724	359.07265	4.59	133 (3), **161** (100), 162(6), 179 (14), 197 (14), 223 (6)	**133** (100)	77 (41), 89 (29), **105** (100), 115 (40), 133 (60)	+	+
**18**	**Nepetoidin A or B**	9.38	C_17_H_13_O_6_^−^	313.07176	313.06826	3.50	**161** (100), 179 (10), 203 (10), 267 (15), 268 (16), 269 (26)	**133** (100)	ND	+	+
**19**	**Clinopodic acid A**	10.04	C_18_H_15_O_7_^−^	343.08233	343.07838	3.95	135 (12), 145 (15), **161** (100), 179 (23), 197 (11), 297 (73), 298 (19)	**133** (100)	ND	+	+
**20**	**Methyl rosmarinate**	10.21	C_19_H_17_O_8_^−^	373.09289	373.08847	4.42	**135** (100), 161 (47), 175 (26), 179 (83), 197 (30), 311 (17), 355 (18)	79 (26), 91 (24), 107 (57), **135** (100)	ND	+	+
***Flavonoid Glycosides and Aglycones***											
**21**	**Quercetin 3-*O*-rutinoside *^a^***	8.17	C_27_H_29_O_16_^−^	609.14611	609.14136	4.75	179 (3), 255 (5), 271 (7), 300 (37), **301** (100), 343 (11), 463 (7)	107 (7), 151 (77), **179** (100), 257 (12), 271 (19), 273 (20)	**151** (100)	+	+
**22**	**Quercetin 3-*O*-glucoside *^a^***	8.42	C_21_H_19_O_12_^−^	463.08820	463.08279	5.41	300 (22), **301** (100)	107 (6), 151 (81), **179** (100), 257 (14), 271 (19), 273 (18)	**151** (100)	+	+
**23**	**Luteolin 7-*O*-hexuronide**	8.50	C_21_H_17_O_12_^−^	461.07255	461.06743	5.12	**285** (100), 286 (14), 327 (3), 357 (4), 381 (3)	151 (36), 175 (86), 199 (68), 217 (67), **241** (100), 243 (56)	185 (32), 197 (53), **198** (100), 199 (85), 213 (33)	+	+
**24**	**Kaempferol 7-*O*-rutinoside**	8.51	C_27_H_29_O_15_^−^	593.15119	593.14990	1.29	229 (3), 257 (4), **285** (100), 286 (12), 327 (3), 547 (11), 548 (3)	197 (26), 213 (30), 229 (52), 241 (43), **257** (100), 267 (52)	163 (62), 185 (13), 213 (21), **229** (100), 239 (27)	+	+
**25**	**Kaempferol 3-*O*-glucoside *^a^***	8.84	C_21_H_19_O_11_^−^	447.09329	447.08843	4.85	255 (19), **284** (100), 285 (85), 327 (20)	227 (14), **255** (100), 256 (21)	211 (67), **227** (100), 255 (10)	+	+
**26**	**Apigenin 7-*O*-glucoside *^a^***	9.11	C_21_H_17_O_11_^−^	445.07764	445.07283	4.80	175 (13), **269** (100), 270 (14), 399 (9), 401 (4)	149 (43), 151 (27), 181 (29), 197 (34), 201 (55), **225** (100)	ND	+	+
**27**	**Thymusin**	11.91	C_17_H_13_O_7_^−^	329.06668	329.06286	3.82	299 (6), **314** (100), 315 (17), 316 (3)	241 (4), 284 (3), 285 (45), 286 (4), 296 (6), **299** (100)	199 (23), 227 (33), 243 (25), 255 (58), **271** (100)	+	+
**28**	**Cirsimaritin *^a^***	12.91	C_17_H_13_O_6_^−^	313.07176	313.06803	3.73	183 (25), 184 (3), 283 (6), 297 (3), **298** (100), 299 (14), 311 (3)	150 (3), 225 (3), 268 (3), 269 (29), 270 (4), **283** (100)	163 (8), 211 (5), 227 (9), 239 (6), **255** (100)	+	+
**29**	**Ladanein**	13.22	C_17_H_13_O_6_^−^	313.07176	313.06810	3.66	183 (46), 184 (6), 198 (3), 283 (5), **298** (100), 299 (15), 311 (4)	269 (20), 270 (3), **283** (100)	163 (9), 211 (5), 227 (12), 239 (5), **255** (100)	+	+
**30**	**Xanthomicrol**	13.55	C_18_H_15_O_7_^−^	343.08233	343.07811	4.22	313 (6), **328** (100), 329 (19), 330 (3)	**313** (100)	193 (5), 270 (13), 285 (38), 295 (24), **298** (100)	+	+
**31**	**Acacetin *^a^***	13.81	C_16_H_11_O_5_^−^	283.06120	283.05807	3.13	**268** (100), 269 (11)	200 (6), 212 (6), 239 (18), 240 (49), **268** (100), 269 (21)	**172** (100), 196 (80), 211 (78), 212 (78), 240 (52)	+	+
**32**	**Kumatakenin**	14.11	C_17_H_13_O_6_^−^	313.07176	313.06784	3.92	183 (59), 184 (8), 185 (3), 198 (5), **298** (100), 299 (16), 311 (5)	255 (12), 270 (19), **283** (100), 284 (8)	**255** (100)	+	+
***Iridoid Glycosides***											
**33**	**Ajugol**	6.21	C_15_H_23_O_9_^−^	347.13476	347.13036	4.40	123 (9), 167 (47), **185** (100), 186 (7), 281 (7), 308 (6), 310 (8)	71 (18), 123 (27), 125 (10), **139** (100), 141 (86), 167 (86)	81 (15), **95** (100), 110 (17), 121 (69), 124 (31)	+	+
**34**	**Bartsioside**	6.24	C_15_H_21_O_8_^−^	329.12419	329.11905	5.14	129 (8), 156 (8), 161 (23), **167** (100), 285 (23), 299 (9)	**149** (100), 123 (10), 121 (15)	ND	+	+
**35**	**Lamiol**	6.34	C_16_H_25_O_10_^−^	377.14532	377.14004	5.28	151 (9), **153** (100), 179 (7), 195 (7), 197 (63), 201 (8), 215 (9)	**109** (100), 135 (7)	ND	–	+
**36**	**Geniposide isomer**	6.68	C_17_H_23_O_10_^−^	387.12967	387.12505	4.62	**179** (100), 180 (13), 207 (11), 225 (50), 258 (8), 340 (14), 341 (33)	146 (22), **161** (100), 164 (68)	**146** (100)	+	+
**37**	**6-*O*-Deoxylamioside**	7.20	C_18_H_27_O_10_^−^	403.16097	403.15625	4.72	137 (47), 181 (21), 191 (21), 199 (48), 247 (20), **343** (100), 361 (73)	113 (4), 119 (5), **137** (100), 181 (44), 199 (55), 299 (3)	80 (3), 107 (3), **119** (100)	+	+
**38**	**Geniposide**	7.50	C_17_H_23_O_10_^−^	387.12967	387.12571	3.96	**161** (100), 163 (3), 207 (5)	105 (3), 133 (95), 143 (3), **146** (100), 161 (5)	**118** (100)	+	+
**39**	**1-*O*-Hexosyl-epideoxyloganic acid**	7.86	C_22_H_33_O_14_^−^	521.18758	521.18257	5.01	153 (18), 197 (92), 341 (50), **359** (100), 475 (34), 476 (29), 477 (23)	109 (11), 135 (7), 153 (17), **197** (100), 315 (6)	87 (5), 109 (22), 135 (6), **153** (100)	+	+
**40**	**Epi-deoxyloganic acid isomer**	7.92	C_16_H_23_O_9_^−^	359.13476	359.12981	4.95	109 (5), 135 (24), 136 (5), 153 (63), 154 (9), **197** (100), 198 (16)	109 (27), 135 (8), **153** (100)	**135** (100)	+	+
**41**	**1,5,9-Epi-deoxyloganic acid*^a^***	8.13	C_16_H_23_O_9_^−^	359.13476	359.12960	5.16	109 (3), 135 (20), 153 (49), **197** (100)	109 (28), 135 (6), **153** (100)	**135** (100)	+	+
**42**	**Boschnaloside**	8.25	C_16_H_23_O_8_^−^	343.13984	343.13512	4.72	101 (7), 113 (15), 143 (5), **161** (100), 181 (12)	71(35), 87(20), **101** (100), 113(51), 143(15)	ND	+	+
**43**	**Deoxyloganetic acid pentoside**	8.26	C_15_H_21_O_8_^−^	329.12419	329.12048	3.71	149 (9), 153 (44), **197** (100), 198 (10), 285 (16)	109(9), 135(7), **153** (100)	107 (11), **135** (100)	+	+
**44**	**Aucubin**	8.43	C_16_H_25_O_8_^−^	345.15549	345.15103	4.46	101 (12), 113 (20), 119 (17), 143 (10), 161 (9), **183** (100)	57 (33), 107 (6), 125 (34), 139 (65), 155 (17), **165** (100)	55 (48), 95 (85), **107** (100), 109 (23), 137 (48)	+	+
**45**	**Nepetariaside**	8.73	C_16_H_27_O_8_^−^	347.17114	347.16679	4.35	101 (40), 113 (47), 119 (50), 143 (16), 167 (75), **185** (100), 329 (17)	57 (5), 139 (85), 141 (10), **167** (100)	57 (92), **109** (100), 124 (3), 125 (5), 137 (7)	+	+
***Other Compounds***											
**46**	**Quinic acid*^a^***	1.59	C_7_H_11_O_6_^−^	191.05611	191.05418	1.93	173 (29), 171 (49), 153 (13), 127 (97), 111 (45), 93 (58), **85** (100)	**57** (100)	ND	+	+
**47**	**3,4-dihydroxyphenethyl alcohol 4-*O*-hexoside**	6.02	C_14_H_19_O_8_^−^	315.10854	315.10459	3.95	123 (8), **153** (100), 154 (7), 269 (4)	109 (5), **123** (100)	81 (11), 93 (12), **95** (100), 105 (46), 123 (6)	+	+
**48**	**12-*O*-HexOsyl-Jasmonate**	7.40	C_18_H_27_O_9_^−^	387.16606	387.16069	5.37	113 (4), 163 (73), 164 (8), **207** (100), 208 (10), 225 (5), 369 (16)	**163** (100)	107 (18), **109** (100), 121 (4), 145 (8), 147 (8)	+	+
**49**	**UmbelLiferone *^a^***	9.33	C_9_H_5_O_3_^−^	161.02442	161.02294	1.48	113 (38), 115 (82), 119 (56), 131 (32), **133** (100), 134 (60), 141 (35)	89 (7), 90 (4), 92 (3), 105 (18), 106 (4), **115** (100)	ND	+	+

*^a^* Confirmed using standards.

**Table 2:** Antimicrobial activities of *N. rtanjensis*, *N. argolica* subsp. *argolica*, and of *cis,trans*-nepetalactone (*cis,trans*-NL), *trans,cis*-nepetalactone (*trans,cis*-NL), 1,5,9-epideoxyloganic acid (1,5,9-*e*DLA) and rosmarinic acid (RA). Values for minimal inhibitory (MIC) and lethal concentration (MBC-bactericidal and MFC-fungicidal concentration) are presented as mg of extract/compound per mL (mg mL^−1^).

Antibacterial Activity	*N. rtanjensis*	*N. argolica*	*cis,trans*-NL	*trans,cis*-NL	RA	1,5,9*-e*DLA	Streptomycin
	MIC-MBC	MIC-MBC	MIC-MBC	MIC-MBC	MIC-MBC	MIC-MBC	MIC-MBC
	[mg mL^−1^]	[mg mL^−1^]	[mg mL^−1^]	[mg mL^−1^]	[mg mL^−1^]	[mg mL^−1^]	[mg mL^−1^]
***B. cereus***	0.2000–0.4000	0.2000–0.4000	0.0400–0.0800	0.0800–0.1200	0.0100–0.0200	0.0600–0.0800	0.1000–0.2000
***S. aureus***	0.1500–0.2000	0.2000-0.4000	0.0100–0.0200	0.0100–0.0400	0.0075–0.0100	0.0050–0.0100	0.0500–0.1000
***M. flavus***	0.2000–0.4000	0.1500–0.4000	0.0300–0.0400	0.0300–0.0400	0.0300–0.040	0.6000–0.8000	0.1000–0.2000
***L. monicytogenes***	0.1500–0.2000	0.2000–0.4000	0.0025–0.0050	0.0100–0.0200	0.0150–0.0200	0.0200–0.0400	0.2000–0.3000
***E. faecalis***	0.4000–0.6000	0.3000–0.4000	0.01500–0.0400	0.0400–0.0800	0.0400–0.0800	0.0300–0.0400	0.1000–0.2000
***P. aeruginosa***	0.1000–0.2000	0.3000–0.4000	0.0100–0.0200	0.0300–0.0400	0.0100–0.0200	0.0400–0.0600	0.2000–0.3000
***E. coli***	0.2000–0.4000	0.1500–0.2000	0.0200–0.0400	0.0200–0.0400	0.0300–0.0400	0.0200–0.0400	0.1000–0.2000
***S. typhimurium***	0.2000–0.4000	0.2000–0.4000	0.0025–0.0050	0.0400–0.0800	0.0300–0.0400	0.0050–0.0100	0.2000–0.3000
**Antifungal Activity**	***N. rtanjensis***	***N. argolica***	***cis,trans*-NL**	***trans,cis*-NL**	**RA**	**1,5,9*-e*DLA**	**Ketoconazole**
	MIC-MFC	MIC-MFC	MIC-MFC	MIC-MFC	MIC-MFC	MIC-MFC	MIC-MFC
	[mg mL^−1^]	[mg mL^−1^]	[mg mL^−1^]	[mg mL^−1^]	[mg mL^−1^]	[mg mL^−1^]	[mg mL^−1^]
***A. fumigatus***	0.1000–0.2000	0.1000–0.2000	0.0100–0.0200	0.0100–0.0200	0.0200–0.0400	0.0400–0.0600	0.2500–0.5000
***A. ochraceus***	0.1000–0.2000	0.1000–0.2000	0.0050–0.0200	0.0025–0.005	0.0050–0.0100	0.0100–0.0200	0.1500–0.2000
***A. versicolor***	0.1000–0.2000	0.1000–0.4000	0.0200–0.0300	0.0050–0.0100	0.0100–0.0200	0.0400–0.0600	0.2000–0.5000
***A. niger***	0.0500–0.1000	0.1000–0.4000	0.0050–0.0200	0.0050–0.0200	0.0075–0.0100	0.0150–0.0400	0.2000–0.5000
***A. funiculosum***	0.2000–0.4000	0.2000–0.4000	0.0200–0.0300	0.0050–0.0100	0.0050–0.0100	0.0050–0.0400	0.2000–0.5000
***P. ochlochloron***	0.2000–0.4000	0.3000–0.4000	0.0050–0.0100	0.0050–0.0100	0.0100–0.0200	0.0600–0.0800	1.0000–1.5000
***P. v. cylopium***	0.3000–0.4000	0.3000–0.4000	0.0100–0.0300	0.0100–0.0200	0.0100–0.0200	0.0600–0.0800	0.2000–0.5000

## Data Availability

The data presented in this study are available on request from the corresponding author.

## Supplementary Materials

The following are available online at https://www.mdpi.com/article/10.3390/ph14050414/s1, Figure S1: Structural characterization of *trans*,*cis*-NL (a) using NMR techniques: 1D (^1^H and ^13^C) (b and c) and 2D (COSY, NOESY and HSQC) (d to f); Supplementary Materials Figure S2: Structural characterization of *cis*,*trans*-NL (a) using NMR techniques: 1D (^1^H and ^13^C) (b and c) and 2D (COSY and HSQC, respectively) (d and e); Supplementary Materials Figure S3: Structural characterization of 1,5,9-epideoxyloganic acid (a), using NMR techniques: 1D (^1^H and ^13^C) (b and c) and 2D (NOESY) (d); Supplementary Materials Figure S4: Proposed structural formula and detailed fragmentation pathway of trihydroxycinnamoylquinic acid (a), boschnaloside (b), deoxyloganetic acid pentoside (c), and 3,4-dihydroxyphenethyl alcohol 4-*O*-hexoside (d).

## References

[1] Salehi B., Valussi M., Jugran A.K., Martorell M., Ramírez-Alarcón K., Stojanović-Radić Z.Z., Antolak H., Kręgiel D., Mileski K.S., Sharifi-Rad M. (2018). Nepeta species: From farm to food applications and phytotherapy. Trends Food Sci. Technol..

[2] Bellahsene C., Bendahou M., Khadir A., Zenati F., Benbelaïd F., Aissaoui N. (2015). Antimicrobial activity and chemical composition of essential oil and hydrosol extract of *Nepeta nepetella* subsp. amethystina (Poir.) Briq. from Algeria. J. Appl. Pharm. Sci..

[3] Kalpoutzakis E., Aligiannis N., Mentis A., Mitaku S., Charvala C. (2001). Composition of the essential oil of two *Nepeta* species and *in vitro* evaluation of their activity against *Helicobacter pylori*. Planta Med..

[4] Nestorović J., Mišić D., Šiler B., Soković M., Glamočlija J., Ćirić A., Maksimović V., Grubišić D. (2010). Nepetalactone content in shoot cultures of three endemic *Nepeta* species and the evaluation of their antimicrobial activity. Fitoterapia.

[5] Formisano C., Rigano D., Senatore F. (2011). Chemical Constituents and Biological Activities of *Nepeta* Species. Chem. Biodiver..

[6] Bates R.B., Sigel C.W. (1963). Terpenoids. Cis-trans- and trans-cis-Nepetalactones. Experientia.

[7] Hardie J., Peace L., Pickett J.A., Smiley D.W.M., Storer J.R., Wadhams L.J. (1997). Sex pheromone stereochemistry and purity affect field catches of male aphids. J. Chem. Ecol..

[8] Peterson C.J., Nemetz L.T., Jones L.M., Coats J.R. (2002). Behavioral activity of catnip (Lamiaceae) essential oil components to the german cockroach (Blattodea: Blattellidae). Entomol. Soc. Am..

[9] Birkett M.A., Hassanali A., Hoglund S., Pettersson J., Pickett J.A. (2011). Repellent activity of catmint, *Nepeta cataria*, and iridoid nepetalactone isomers against Afro-tropical mosquitoes, ixodid ticks and red poultry mites. Phytochemistry.

[10] Gkinis G., Tzakou O., Iliopoulou D., Roussis V. (2003). Chemical Composition and Biological Activity of *Nepeta parnassica* Oils and Isolated Nepetalactones. Z. Naturforsch. C. J. Biosci..

[11] Kumar V., Mathela C.S., Tewari G., Singh D. (2014). Antifungal activity of *Nepeta elliptica* Royle ex Benth. oil and its major constituent (7R)-trans,trans-nepetalactone: A comparative study. Ind. Crop. Prod..

[12] Murai F., Tagawa M., Damtoft S., Jensen S.R., Nielsen B.J. (1984). (1*R*, *5R*, *8S, 9S)-*Deoxyloganic acid from *Nepeta cataria*. Chem. Pharm. Bull..

[13] Takeda Y., Ooiso Y., Masuda T., Honda G., Otsuka H., Sezik E., Yesilada E. (1998). Iridoid and eugenol glycosides from *Nepeta cadmea*. Phytochemistry.

[14] Kökdil G., Yalçin S.M., Topçu G. (1999). Nepetalactones and other constituents of *Nepeta nuda* ssp. *albiflora*. Turk. J. Chem..

[15] Ahmed A.A., Hassan H.E., Hegazy M.F., Tzakou O., Couladis M., Mohamed A.E.-H.H., Abdella M.A., Pare P. (2006). Argolic acid A and argolic methyl ester B, two new cyclopentano-monoterpenes diol from *Nepeta argolica*. Nat. Prod. Com..

[16] Stojanović G., Radulović N., Lazarević J., Miladinović D., Đoković D. (2005). Antimicrobial Activity of *Nepeta rtanjensis* Essential Oil. J. Essent. Oil Res..

[17] Mišić D., Šiler B., Gašić U., Avramov S., Živković S., Nestorović Živković J., Milutinović M., Tešić Ž. (2015). Simultaneous UHPLC/DAD/(+/−)HESI-MS/MS analysis of phenolic acids and nepetalactones in methanol extracts of *Nepeta* species: A possible application in chemotaxonomic studies. Phytochem. Anal..

[18] Aničić N., Matekalo D., Skorić M., Živković J.N., Petrović L., Dragićević M., Dmitrović S., Mišić D. (2020). Alterations in nepetalactone metabolism during polyethylene glycol (PEG)-induced dehydration stress in two *Nepeta* species. Phytochemistry.

[19] Sharma A., Cooper R., Bhardwaj G., Cannoo D.S. (2020). The genus *Nepeta*: Traditional uses, phytochemicals and pharmacological properties. J. Ethnopharmacol..

[20] Wang M., Cheng K.W., Wu Q., Simon J.E. (2007). Quantification of nepetalactones in catnip (*Nepeta cataria* L.) by HPLC coupled with ultraviolet and mass spectrometric detection. Phytochem. Anal..

[21] Goldansaz S.M., Festa C., Pagano E., De Marino S., Finamore C., Parisi O.A., Borrelli F., Sonboli A., D’Auria M.V. (2019). Phytochemical and Biological Studies of *Nepeta asterotricha* Rech. f. (Lamiaceae): Isolation of Nepetamoside. Molecules.

[22] Jamzad Z., Chase M.W., Ingrouille M., Simmonds M.S.J., Jalili A. (2003). Phylogenetic relationships in *Nepeta* L. (Lamiaceae) and related genera based on ITS sequence data. Taxon.

[23] Fraga B.M., González-Coloma A., Alegre-Gómez S., López-Rodríguez M., Amador L.J., Díaz C.E. (2017). Bioactive constituents from transformed root cultures of *Nepeta teydea*. Phytochemistry.

[24] Miceli N., Taviano M.F., Giuffrida D., Trovato A., Tzakou O., Galati E.M. (2005). Anti-inflammatory activity of extract and fractions from *Nepeta sibthorpii* Bentham. J. Ethnopharmacol..

[25] Kumar V., Sood H., Sharma M., Chauhan R.S. (2013). A proposed biosynthetic pathway of picrosides linked through the detection of biochemical intermediates in the endangered medicinal herb *Picrorhiza kurroa*. Phytochem. Anal..

[26] Huang X.H., Chen J., Xu X.Q., Zhang W.T., Zhao C.C. (2016). A new phenolic compound from *Schizonepeta tenuifolia*. Chem. Nat. Compd..

[27] Aničić N., Matekalo D., Skorić M., Pećinar I., Brkušanin M., Nestorović Živković J., Dmitrović S., Dajić Stevanović Z., Schulz H., Mišić D. (2018). Trichome-specific and developmentally regulated biosynthesis of nepetalactones in leaves of cultivated *Nepeta rtanjensis* plants. Ind. Crops Prod..

[28] Xu Z., Xie J., Soteyome T., Peters B.M., Shirtliff M.E., Liu J., Harro J.M. (2019). Polymicrobial interaction and biofilms between *Staphylococcus aureus* and *Pseudomonas aeruginosa*: An underestimated concern in food safety. Curr. Opin. Food Sci..

[29] Smiljković M., Dias M.I., Stojković D., Barros L., Bukvički D., Ferreira I.C.F.R., Soković M. (2018). Characterization of phenolic compounds in tincture of edible *Nepeta nuda*: Development of antimicrobial mouthwash. Food Funct..

[30] Mousavi Nia A., Kalantaripour T.P., Basiri M., Vafaee F., Asadi-Shekaari M., Eslami A., Zadeh F.D. (2017). *Nepeta dschuparensis* Bornm. extract moderates COX-2 and IL-1β proteins in a rat model of cerebral ischemia. Iran. J. Med. Sci..

[31] Skorić M., Gligorijević N., Čavić M., Ristić M., Mišić D., Radulović S. (2017). Cytotoxic activity of *Nepeta rtanjensis* Diklić and Milojević essential oil and and its mode of action. Ind. Crop. Prod..

[32] Banjanac T., Dragićević M., Šiler B., Gašić U., Bohanec B., Nestorović Živković J., Trifunović S., Mišić D. (2017). Chemodiversity of two closely related tetraploid *Centaurium* species and their hexaploid hybrid: Metabolomic search for high-resolution taxonomic classifiers. Phytochemistry.

[33] Čolić S.D., Fotirić Akšić M.M., Lazarević K.B., Zec G.N., Gašić U.M., Dabić Zagorac D., Natić M.M. (2017). Fatty acid and phenolic profiles of almond grown in Serbia. Food Chem..

[34] Kartsev V., Lichitsky B., Geronikaki A., Petrou A., Smiljkovic M., Kostic M., Radanovic O., Soković M. (2019). Design, synthesis and antimicrobial activity of usnic acid derivatives. MedChemComm.

[35] Soković M., van Griensven L.J.L.D. (2006). Antimicrobial activity of essential oils and their components against the three major pathogens of the cultivated button mushroom, *Agaricus bisporus*. Eur. J. Plant Pathol..

[36] Sirakanyan S., Kartsev V., Spinelli D., Geronikaki A., Petrou A., Ivanov M., Glamočlija J., Soković M., Hakobyan E., Hovakimyan A. (2021). Synthesis and antimicrobial activity of new 2-piperazin-1-yl-N-1,3-thiazol-2-ylacetamides of cyclopenta[c]pyridines and pyrano[3,4-c]pyridines. Archiv Der Pharmazie.

[37] Nikolić M.M., Jovanović K.K., Marković T.L., Marković D.L., Gligorijević N.N., Radulović S.S., Kostić M., Glamočlija J.M., Soković M.D. (2017). Antimicrobial synergism and cytotoxic properties of *Citrus limon* L., *Piper nigrum* L. and *Melaleuca alternifolia* (Maiden and Betche) Cheel essential oils. J. Pharm. Pharmacol..

[38] Jevtić B., Djedović N., Stanisavljević S., Gašić U., Mišić D., Despotović J., Samardžić J., Miljković D., Timotijević G. (2017). Anti-encephalitogenic effects of cucumber leaf extract. J. Funct. Foods.

