# Supplementary material for: Antimicrobial and Immunomodulating Activities of Two Endemic Nepeta Species and Their Major Iridoids Isolated from Natural Sources
Source: Pharmaceuticals (Basel). 2021 Apr 28;14(5):414. doi: 10.3390/ph14050414 (PMC8145025; doi:10.3390/ph14050414)
Supplement: Supplementary file 1 [file pharmaceuticals-14-00414-s001.zip › pharmaceuticals-1186407-supplementary.pdf]

Supplementary material

Article

Antimicrobial and immunomodulating activities of two endemic *Nepeta* species and their major iridoids isolated from natural sources

Neda Aničić [1], Uroš Gašić [1,*], Feng Lu [2], Ana Ćirić [1], Marija Ivanov [1], Bojan Jevtić [3], Milena Dimitrijević [4], Boban Anđelković [5], Marijana Skorić [1], Jasmina Nestorović Živković [1], Yingle Mao [2], Jia Liu [2], Chunping Tang [2], Marina Soković [1], Yang Ye [2], Danijela Mišić [1,2,*]

[1] Department of Plant Physiology, Institute for Biological Research "Siniša Stanković"- National Institute of Republic of Serbia, University of Belgrade, Bulevar despota Stefana 142, 11060 Belgrade, Serbia; neda.anicic@ibiss.bg.ac.rs (N.A.); rancic@ibiss.bg.ac.rs (A.Ć.); marija.smiljkovic@ibiss.bg.ac.rs (M.I.); mdevic@ibiss.bg.ac.rs (M.S.); jasmina.nestorovic@ibiss.bg.ac.rs (J.N.Ž.); mris@ibiss.bg.ac.rs (M.S.)

[2] State Key Laboratory of Drug Research and Department of Natural Product Chemistry, Shanghai Institute of Materia Medica, Chinese Academy of Sciences, 555 Zu-Chong-Zhi Road, Zhangjiang Hi-Tech Park, Shanghai 201203, People's Republic of China; fenglu@simm.ac.cn (F.L.); maoyingle@simm.ac.cn (Y.M.); jia.liu@simm.ac.cn (J.L.); tangcp@simm.ac.cn (C.T.); yye@simm.ac.cn (Y.Y.)

[3] Department of Immunology, Institute for Biological Research "Siniša Stanković"- National Institute of Republic of Serbia, University of Belgrade, Bulevar despota Stefana 142, 11060 Belgrade, Serbia; bojanbh@gmail.com (B.J.)

[4] Center of Excellence for Green Technologies, Institute for Multidisciplinary Research, University of Belgrade, Kneza Višeslava 1, 11030 Belgrade, Serbia; milena.dimitrijevic@imsi.rs (M.D.)

[5] Faculty of Chemistry, University of Belgrade, P.O. Box 51, 11158, 11000 Belgrade, Serbia; aboban@chem.bg.ac.rs (B.A.)

* Correspondence: uros.gasic@ibiss.bg.ac.rs (U.G.); dmisic@ibiss.bg.ac.rs (D.M.); Tel.: +381112078385 (D.M.)

Supplementary Figure captions

Figure S1. Structural characterization of *trans,cis*-NL (a) using NMR techniques: 1D (^{1}H and ^{13}C) (b and c) and 2D (COSY, NOESY and HSQC) (d to f).

Figure S2. Structural characterization of *cis,trans*-NL (a) using NMR techniques: 1D (^{1}H and ^{13}C) (b and c) and 2D (COSY and HSQC, respectively) (d and e);.

Figure S3. Structural characterization of 1,5,9-epideoxyloganic acid (a), using NMR techniques: 1D (^{1}H and ^{13}C) (b and c) and 2D (NOESY) (d).

Figure S4. Proposed structural formula and detailed fragmentation pathway of trihydroxycinnamoylquinic acid (a), boschnaloside (b), deoxyloganetic acid pentoside (c), 3,4-dihydroxyphenethyl alcohol 4-*O*-hexoside (d).

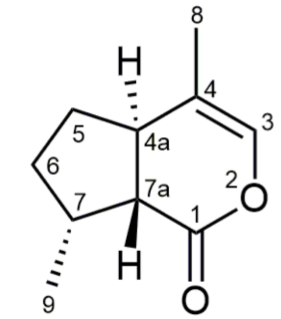

Figure S1a. Chemical structure of *trans, cis* nepetalactone.

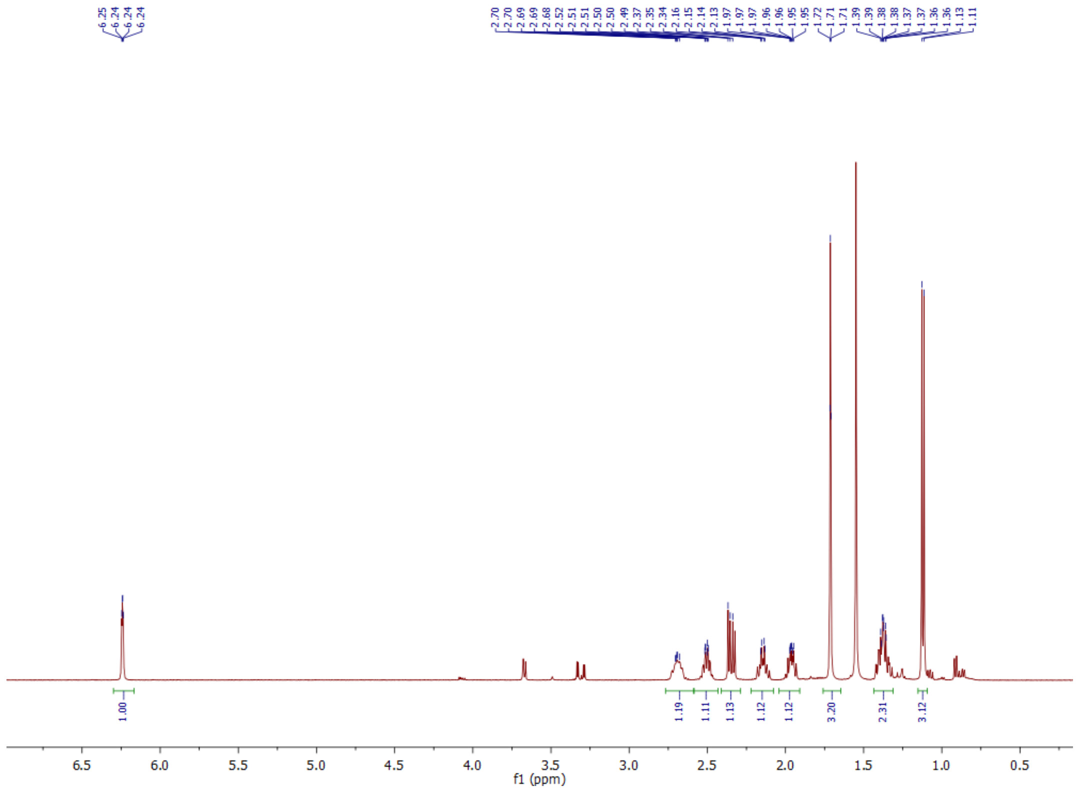

Figure S1b. ^{1}H-NMR spectrum (500 MHz) of *trans, cis* nepetalactone in chloroform –*d*.

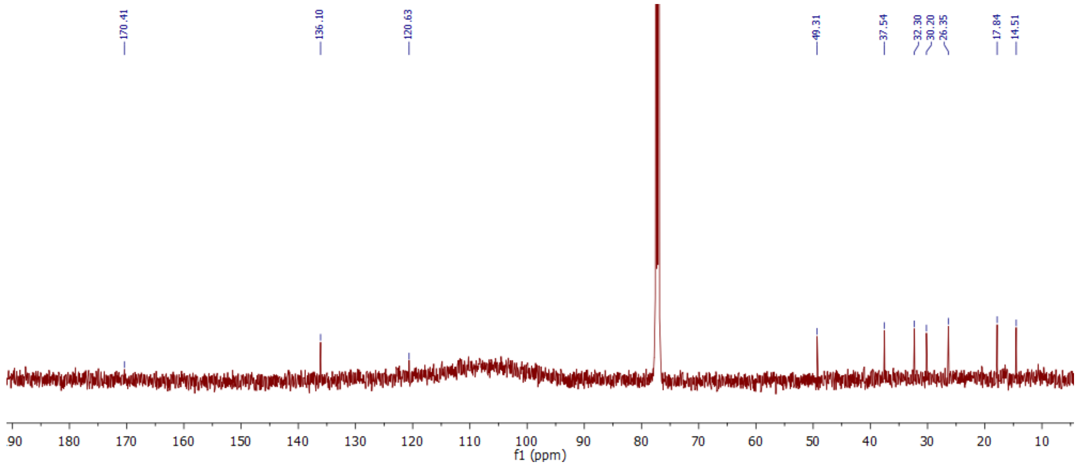

Figure S1c. ^{13}C-NMR spectrum (125 MHz) of *trans, cis* nepetalactone in chloroform –*d*.

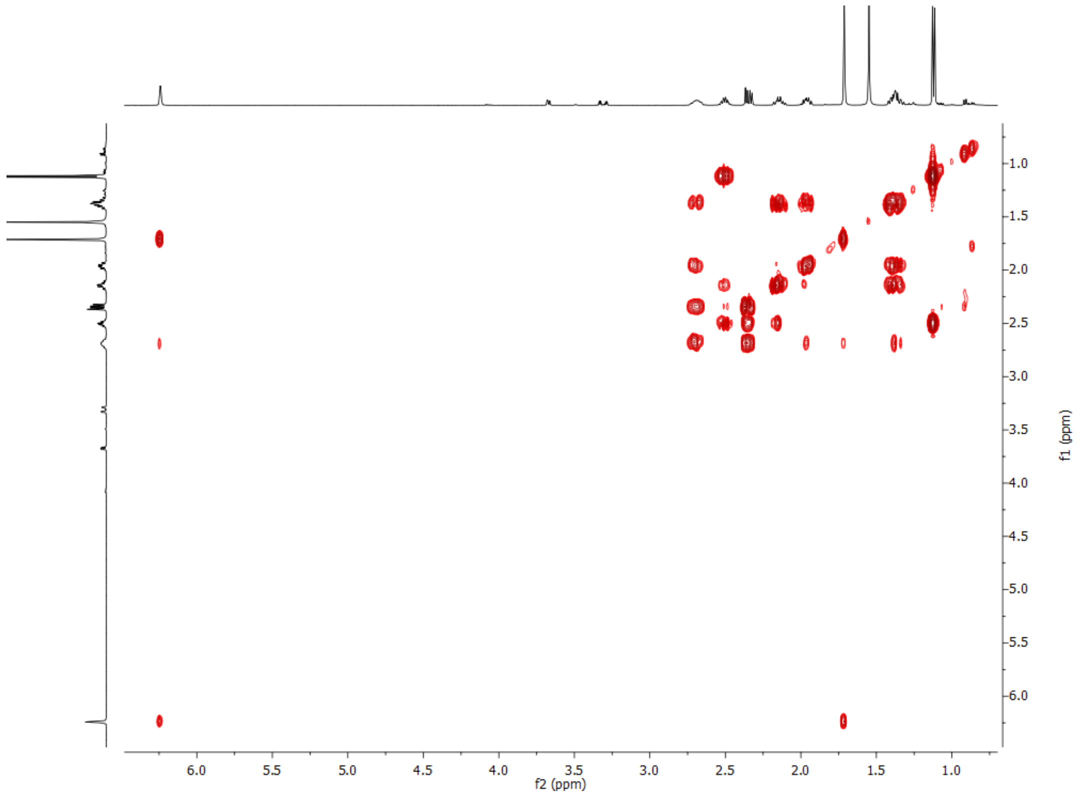

Figure S1d. COSY spectrum of *trans, cis* nepetalactone in chloroform –*d*.

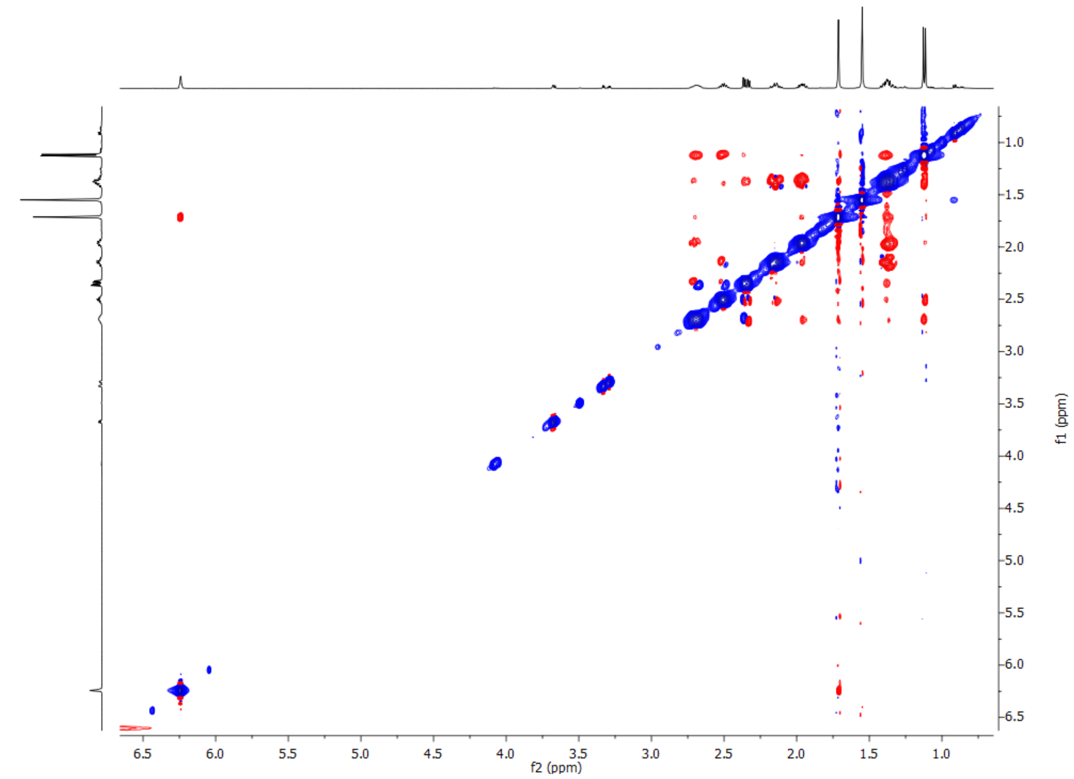

Figure S1e. NOESY spectrum of *trans, cis* nepetalactone in chloroform –*d*.

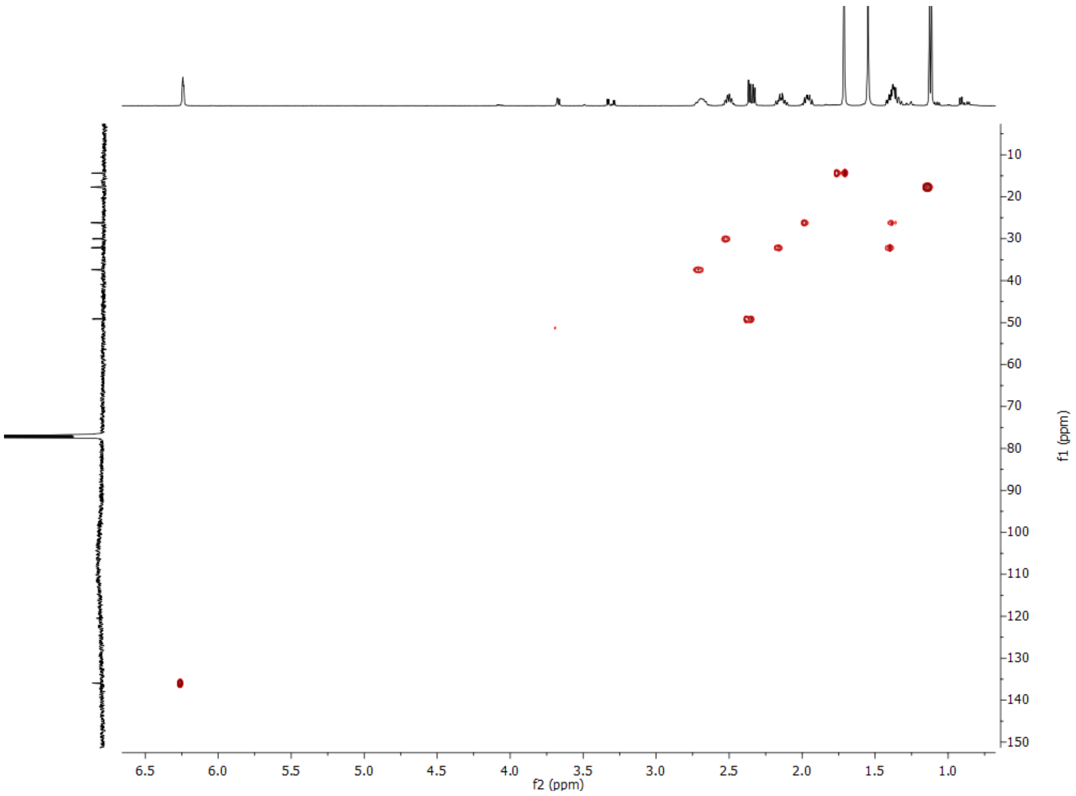

Figure S1f. HSQC spectrum of *trans, cis* nepetalactone in chloroform –*d*.

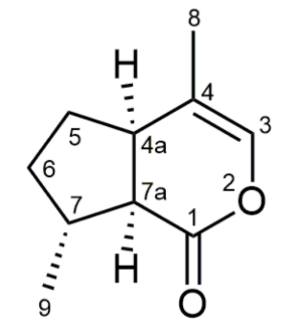

Figure S2a. Chemical structure of *cis,trans* nepetalactone.

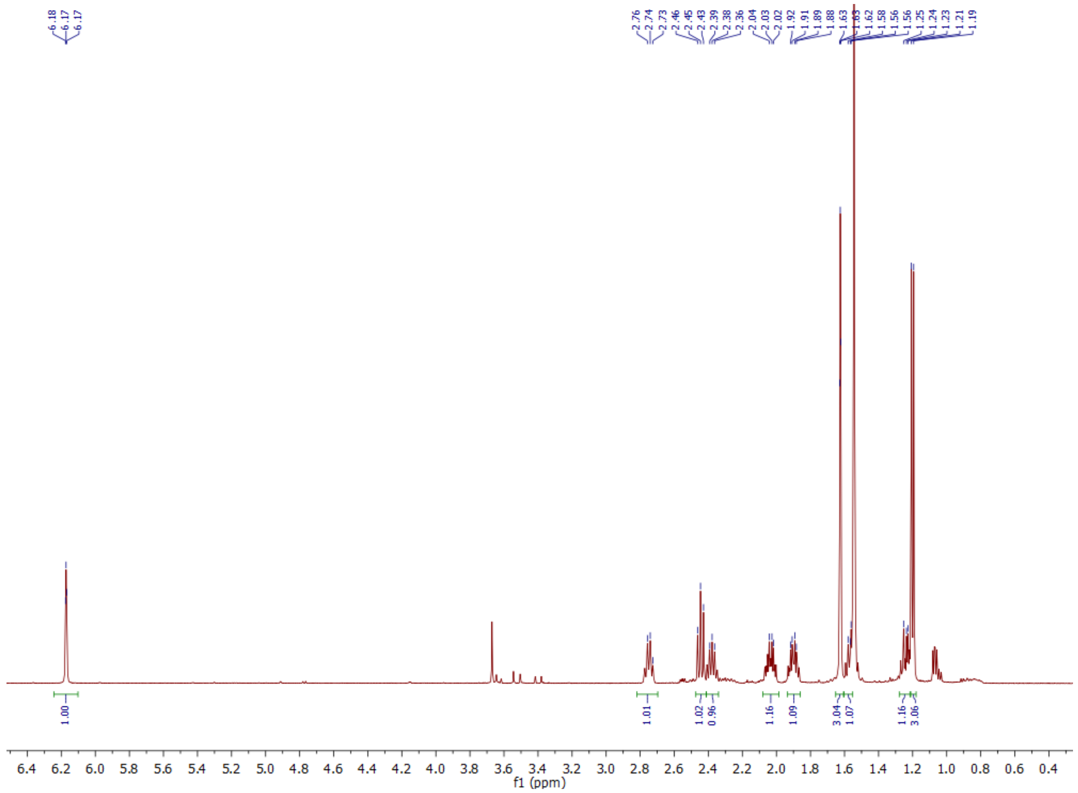

Figure S2b.^{1}H-NMR spectrum (500 MHz) of *cis, trans* nepetalactone in chloroform –*d*.

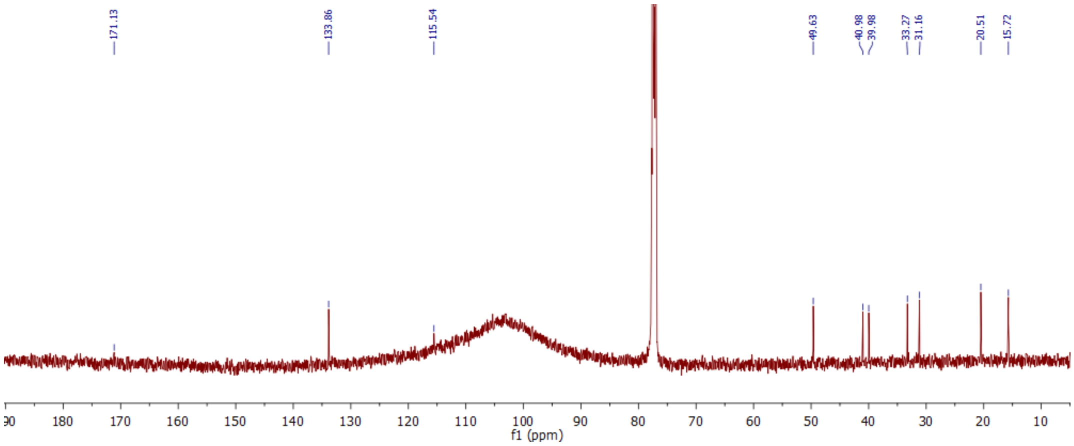

Figure S2c. ^{13}C-NMR spectrum (125 MHz) of *cis, trans* nepetalactone in chloroform –*d*.

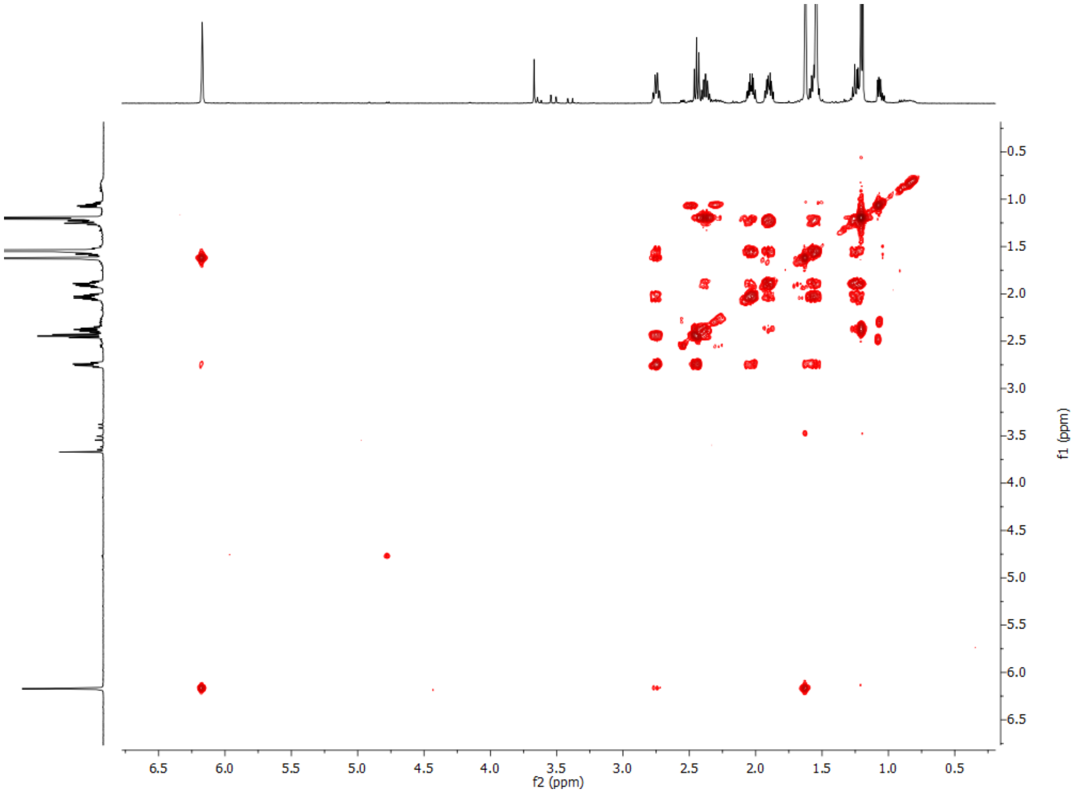

Figure S2d. H-H COSY spectrum of *cis, trans* nepetalactone in chloroform –*d*.

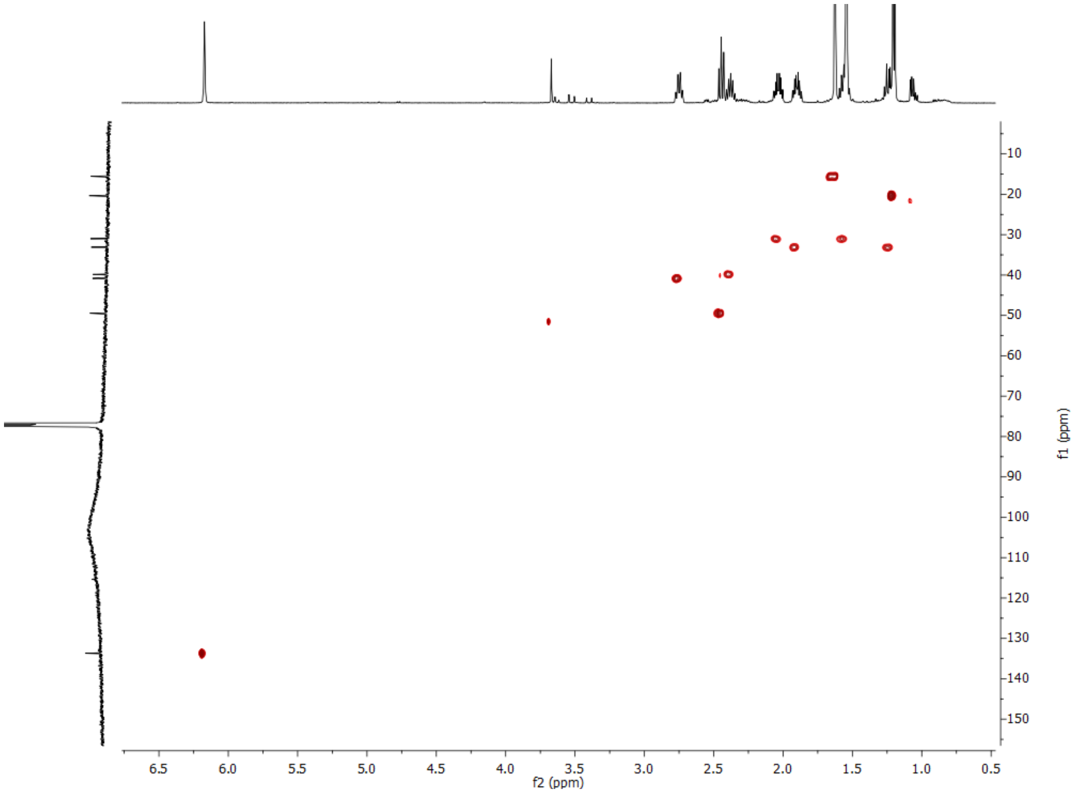

Figure S2e. HSQC spectrum of *cis, trans* nepetalactone in chloroform –*d*.

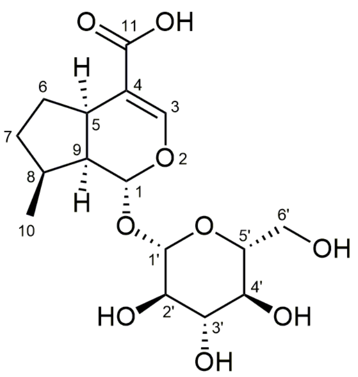

Figure S3a. Chemical structure of 1,5,9-epideoxyloganic acid.

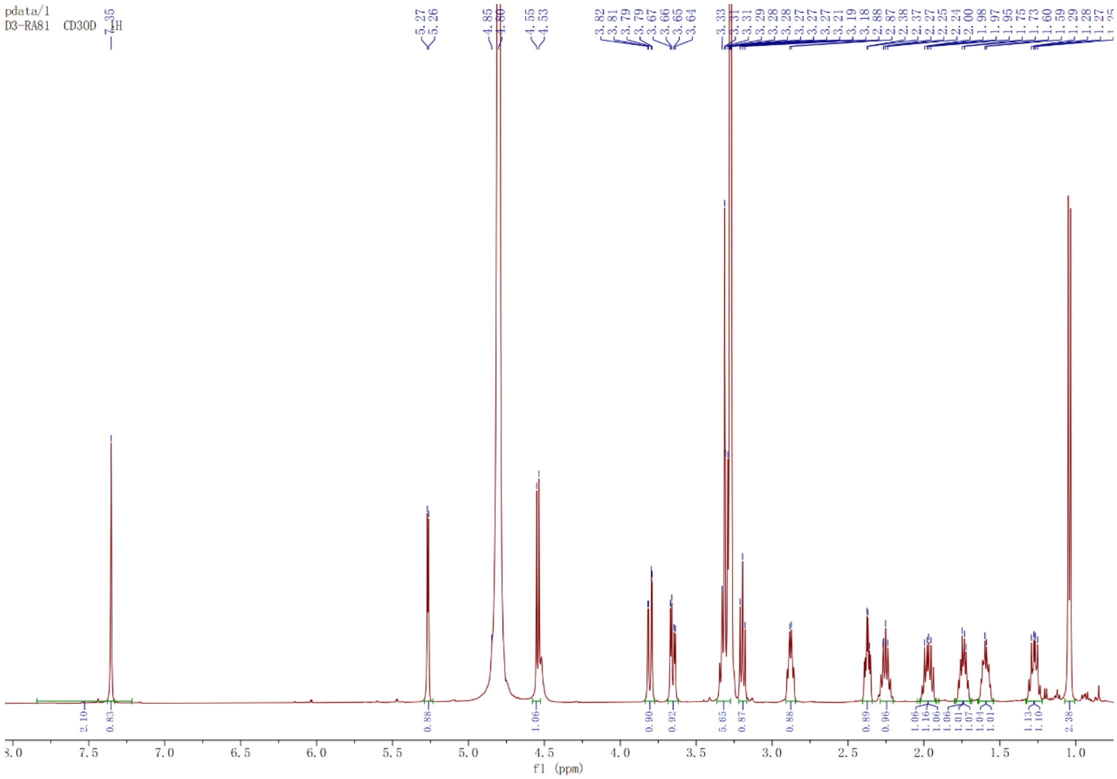

Figure S3b. ^{1}H-NMR spectrum (500 MHz) of 1,5,9-epideoxyloganic acid in methanol-d_4

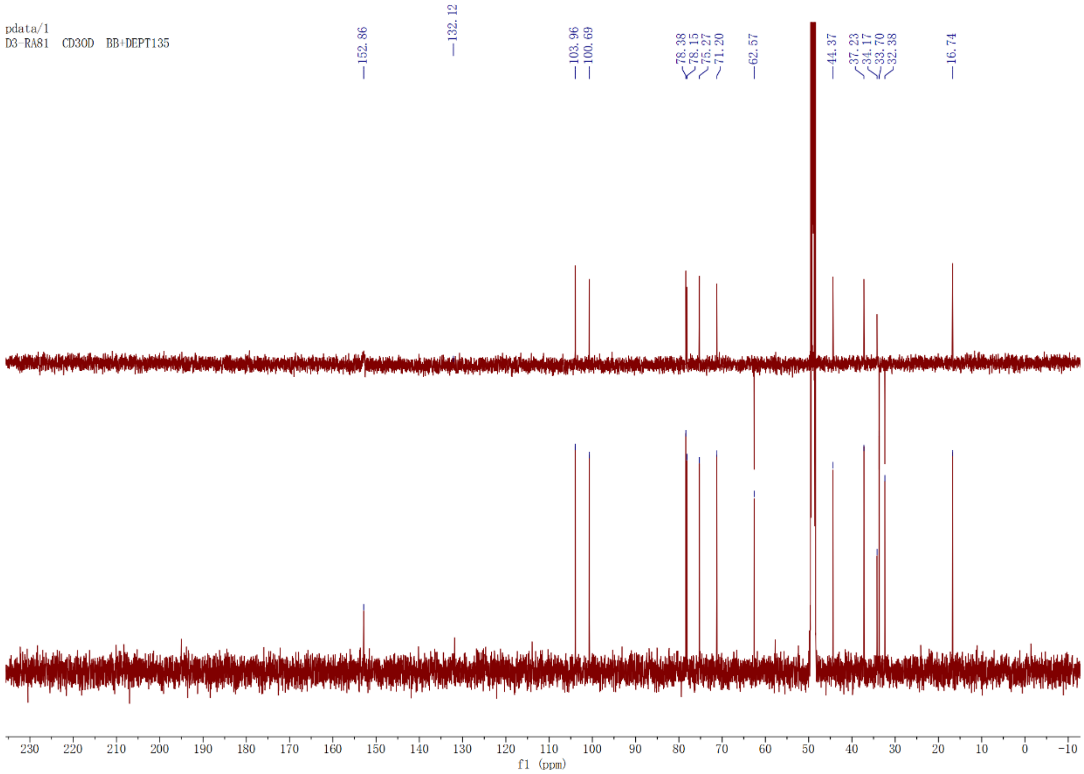

Figure S3c. ^{13}C-NMR spectrum (125 MHz) of 1,5,9-epideoxyloganic acid in methanol-d_4

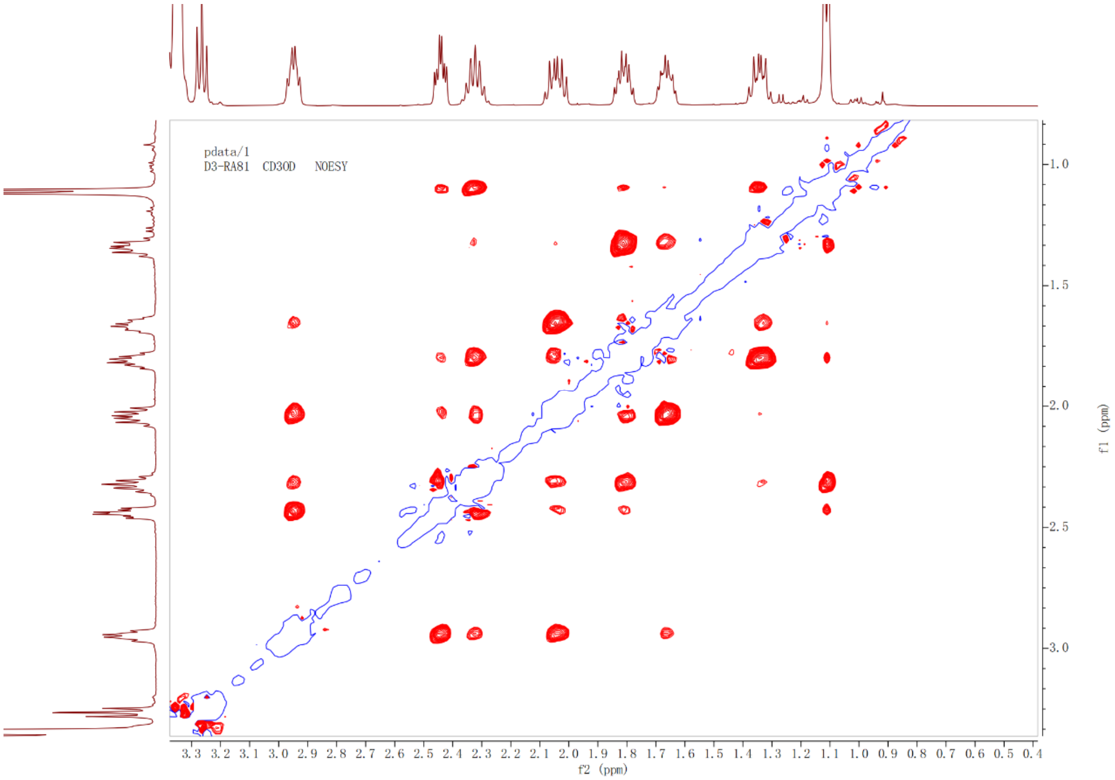

Figure S3d. NORSY spectrum (500 MHz) of 1,5,9-epideoxyloganic acid in methanol-d_4

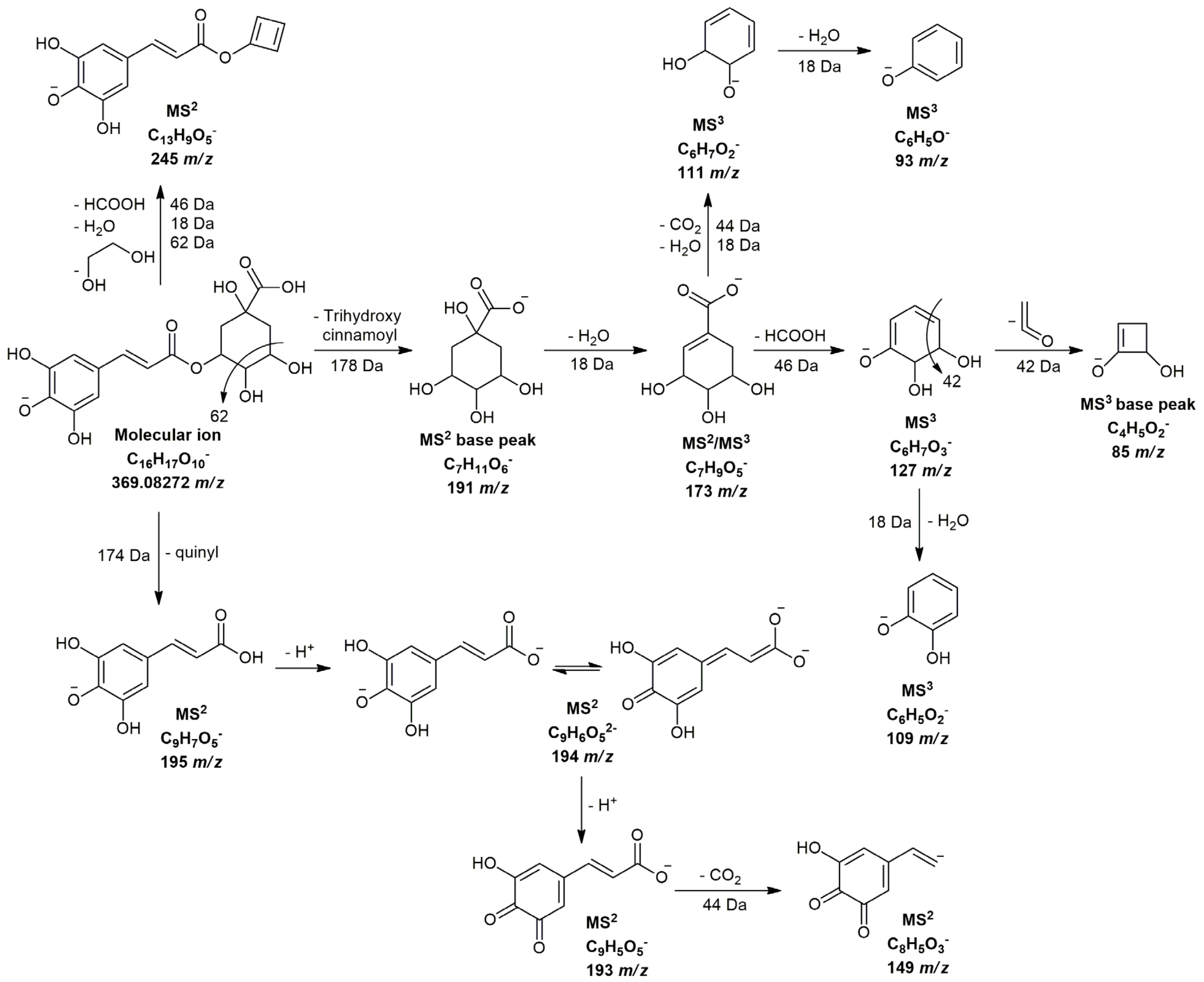

Figure S4a. Proposed structural formula of trihydroxycinnamoylquinic acid (compound **8**), as well as its detailed fragmentation pathway.

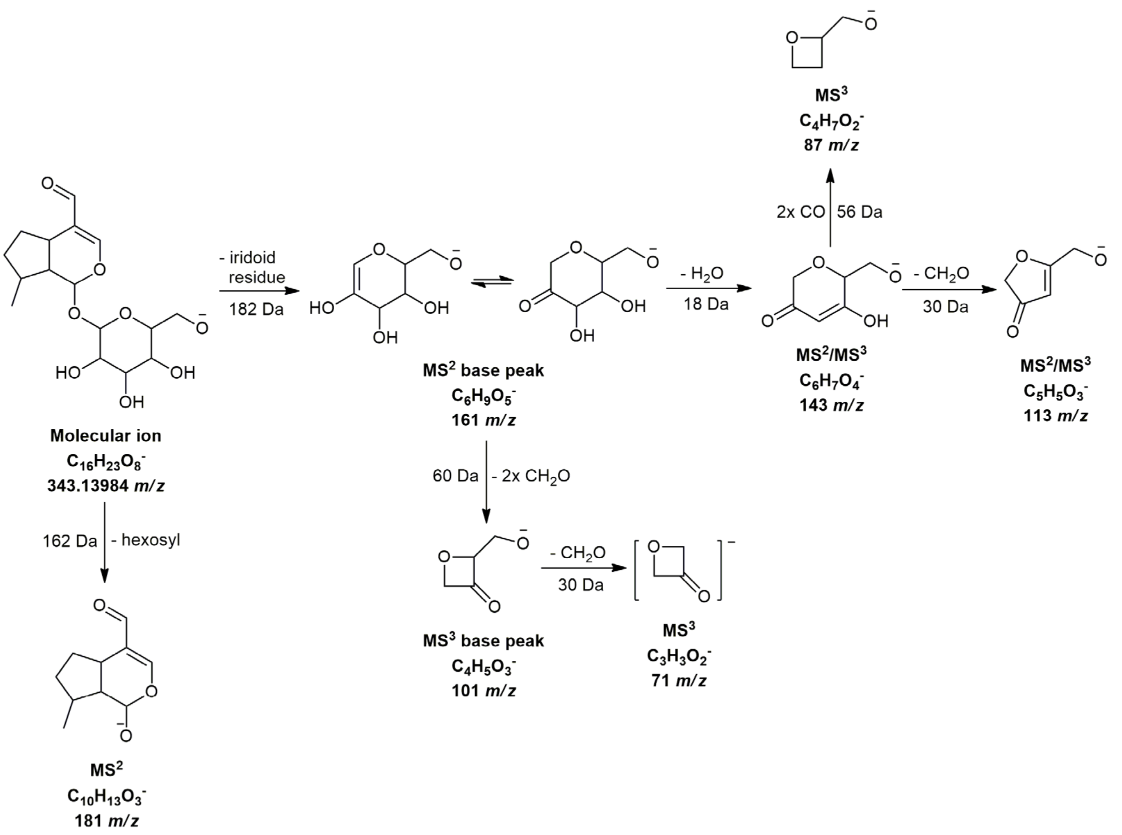

Figure S4b. Proposed fragmentation pathway of boschnaloside (compound **42**).

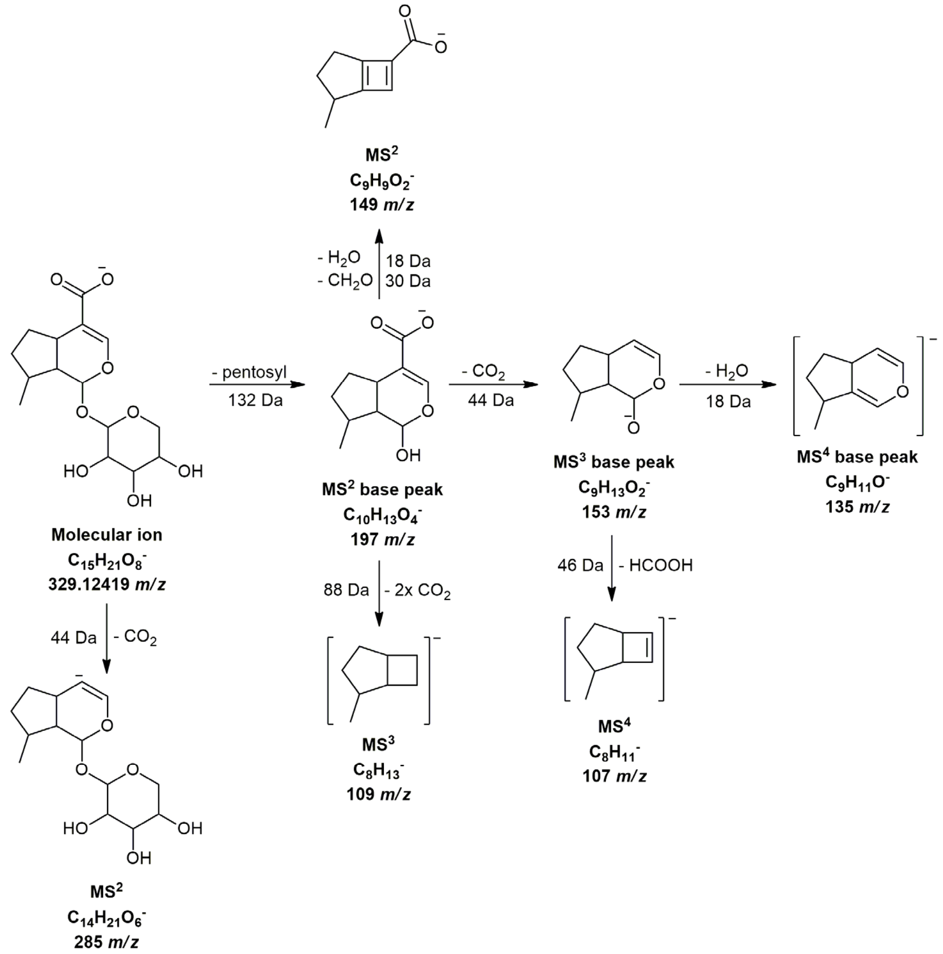

Figure S4c. Proposed structure and detailed fragmentation pathway deoxyloganetic acid pentoside (compound **43**).

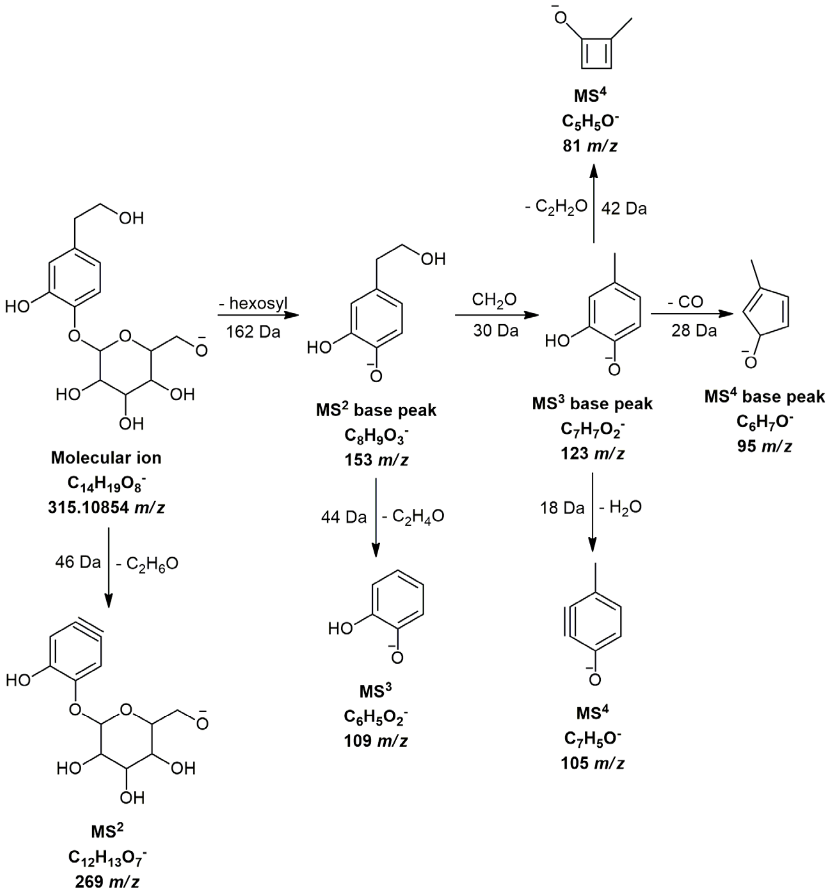

Figure S4d. Fragmentation pathway of 3,4-dihydroxyphenethyl alcohol 4-*O*-hexoside (compound **47**).
